# Evolution and conserved functionality of organ size and shape regulator PEAPOD

**DOI:** 10.1371/journal.pone.0263928

**Published:** 2022-02-11

**Authors:** Ruth Cookson, Somrutai Winichayakul, Hong Xue, Kim Richardson, Roger Moraga, Aurelie Laugraud, Ambarish Biswas, Greg Bryan, Nick Roberts

**Affiliations:** 1 Plant Biotechnology, Grasslands Research Centre, AgResearch Ltd., Palmerston North, New Zealand; 2 Bioinformatics and Statistics, Grasslands Research Centre, AgResearch Ltd., Palmerston North, New Zealand; Universidad Miguel Hernández de Elche, SPAIN

## Abstract

Transcriptional regulator PEAPOD (PPD) and its binding partners comprise a complex that is conserved throughout many core eudicot plants with regard to protein domain sequence and the function of controlling organ size and shape. Orthologues of PPD also exist in the basal angiosperm *Amborella trichopoda*, various gymnosperm species, the lycophyte *Selaginella moellendorffii* and several monocot genera, although until now it was not known if these are functional sequences. Here we report constitutive expression of orthologues from species representing diverse taxa of plant phylogeny in the Arabidopsis Δ*ppd* mutant. PPD orthologues from *S*. *moellendorffii*, gymnosperm *Picea abies*, *A*. *trichopoda*, monocot *Musa acuminata*, and dicot *Trifolium repens* were able to complement the mutant and return it to the wild-type phenotype, demonstrating the conserved functionality of PPD throughout vascular plants. In addition, analysis of bryophyte genomes revealed potential PPD orthologues in model liverwort and moss species, suggesting a more primitive lineage for this conserved regulator. The Poaceae (grasses) lack the genes for the PPD module and the reason for loss of the complex from this economically significant family is unclear, given that grasses were the last of the flowering plants to evolve. Bioinformatic analyses identified putative PPD orthologues in close relatives of the Poaceae, indicating that the explanation for absence of PPD in the grasses may be more complex than previously considered. Understanding the mechanisms which led to loss of PPD from the grasses will provide insight into evolution of the Poaceae.

## Introduction

The production of new organs throughout the life cycle of a plant is an ongoing and flexible process. This plasticity utilizes pluripotent cells which receive signals via specific genetic switches to co-ordinate the temporal and spatial division, differentiation, and expansion of the cells. In the simplest scenario, transcription factors control and co-ordinate the expression of a range of genes involved in a common process or pathway while transcriptional regulators enable or suppress the functionality of transcription factors. It is more recently accepted however, that the switches are a complex network of transcription factors and transcriptional regulators acting in a combinatorial manner enabling both flexibility and specificity over target gene regulation [1,2].

While many of the 50–60 transcription factor gene families present in flowering plants exist in basal land plants, the average size of these families is considerably larger in the angiosperms and is likely linked to gene duplication [3]. Combined with the fact that only 15–30 of the transcription factor gene families are found in chlorophyte algae, it appears there was a large increase in transcription factor families early in the evolution of land plants (embryophytes) [3].

The *TIFY* gene family is one such example of transcription factors that are associated specifically with the emergence of the land plants and where the expansion of the gene family correlates with the increasing complexity in structure and development of embryophytes [4,5]. *TIFY* genes code for a conserved amino acid pattern which was originally called a ZIM (Zinc-finger protein expressed in inflorescence meristem) domain but was re-named *TIFY* to highlight the most conserved amino acid pattern (TIF[F/Y]XG) [5]. There are four *TIFY* subfamilies; these are labelled with the most characteristic other domain present on the protein: TIFY, Jasmonate ZIM-domain (JAZ); PEAPOD (PPD) and ZIM-like (ZML) [4]. The *TIFY* subfamily only has a TIFY domain while *JAZ* contains the TIFY domain and the Jas domain; *PPD* contains both PPD and TIFY domains as well as a truncated Jas domain (Jas*); and *ZML* contains TIFY, CCT and ZML domains [4,6]. The TIFY family in Arabidopsis consists of 18 proteins categorized into two groups by the presence or absence of a C2C2-GATA zinc finger; the ZML proteins are in group I while the remaining subfamilies are in group II since they lack this motif [5].

The PPD class of TIFY genes regulate leaf morphology in two ways: by controlling arrest of pavement cell divisions, known as the primary arrest front [7,8] and via regulating meristemoid (precursors of stomata) proliferation [9,10], which also affects stomatal density. Additional roles for PPD have been demonstrated in vascular development [9,11,12], flowering time [11,13], maturation of flowers, fruit and seeds [9,14–17] and regulation of hormone and light signaling processes [10,11] (reviewed in [18]).

In Arabidopsis, *PPD1* and *PPD2* constitute a homologous gene pair with redundant function [9] although differential expression patterns in discrete plant organs [12] suggest that PPD1 and 2 function through distinct pathways in addition to sharing a mutual pathway. Recent evidence indicates that PPD2 is the major interactor throughout the development of leaves and seeds [12,13]. PPD proteins interact with various polypeptide binding partners to form transcriptional repressor complexes (recently comprehensively reviewed by [18]). In short, KINASE-INDUCIBLE DOMAIN INTERACTING PROTEIN (KIX) 8, KIX9 and NOVEL INTERACTOR OF JAZ (NINJA) are recruited to the complex by PPD to regulate target genes [10]. NINJA is a well-known adaptor protein which recruits the generic repressor protein TOPLESS (TPL) to the complex [19]. The F-box protein STERILE APETALA F(SAP) regulates the PPD complex by physically interacting with PPD and KIX proteins and targeting them for degradation by the E3 ubiquitin ligase complex [14,15]. TIFY8 has also been identified as a binding partner of PPD and analogous with the PPDs, TIFY8 is able to bind NINJA and recruit TPL to a repressor complex [20]. Target genes of PPD include two D-type cyclin genes (cell cycle regulators of the G1-S phase transition [21]) [10] and the influence of PPD on leaf shape is partially exerted by negative regulation of these cyclin genes [7].

**Fig 1:**
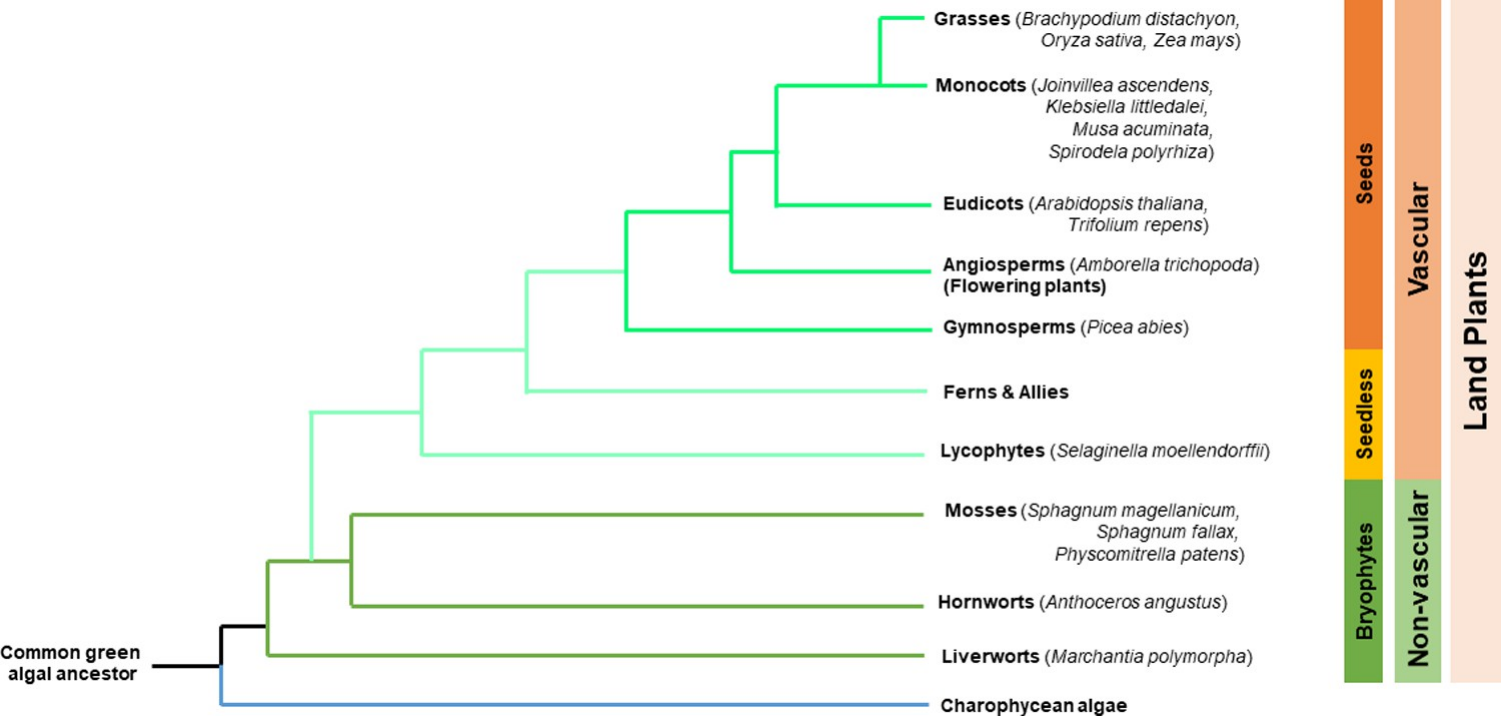
Simplified diagram outlining current understanding of plant phylogeny, including species discussed in this publication. Hornworts, Liverworts and Mosses make up the bryophytes, non-vascular land plants which reproduce via spores. Lycophytes are vascular spore producers and have structures called microphylls rather than true leaves. The dicot *Amborella trichopoda* is considered a representative basal angiosperm, with a primitive form of xylem tissue compared to other flowering plants.

PPD orthologues are present in all vascular plants with published genomes, including the basal angiosperm *Amborella trichopoda* [12], the lycophyte *Selaginella moellendorfii* [4] and monocots such as banana (*Musa acuminata*), oil palm (*Elaeis guineensis*) [12] and duckweed (*Spirodela polyrhiza*) [22] (Fig 1). However, orthologues of PPD and binding partners KIX8/9 and SAP appear to be absent from Poaceae, the family of grasses [9,18]. PPD’s role in leaf development and stomatal patterning implies that these processes differ sufficiently in grasses to explain the absence of the PPD/KIX/SAP module, however the complete reason remains elusive. Although orthologues of many leaf and stomatal development genes exist in grasses [23–25], differences in leaf morphology and stomatal patterning set the grasses apart from dicots and non-grass monocots. Grasses share the leaf traits of parallel venation and stomata on abaxial and adaxial leaf surfaces with other monocot species such as banana [26]. However, the stomata of grasses differ from other plant families, containing dumbbell shaped guard cells arranged in ‘files’ of cells rather than the kidney shaped guard cells scattered across the leaf surface of dicots and non-grass monocots [24]. Grass stomata are formed by the asymmetric division of an epidermal cell file located next to a leaf vein rather than by self-renewing meristemoids as in dicots [27].

Given the recent evolution of the Poaceae as a distinct taxonomic group, we sought to investigate the evolutionary significance of their lack of *PPD* and the conservation of *PPD* throughout vascular plants. Here we report preservation of protein domain sequence, and demonstrate functionality, of PPD orthologues from species representing different nodes of the evolutionary tree.

## Material and methods

### Plant materials and growth conditions

*Arabidopsis thaliana* (L.) Heynh ecotype L*er* was used as wild-type (WT). The *Δppd* loss of function deletion mutant (with PPD1 and PPD2 deleted) was as previously described in [9]. Plants were grown in a temperature-controlled glasshouse at a continuous 21°C or in a controlled environment cabinet at 23°C and 16-h photoperiod.

### Cloning of gene constructs

*PPD* gene sequences from a range of species were synthesized with flanking attL sites (GenScript Biotech, China) and cloned into expression cassettes to enable over expression from the Cauliflower Mosaic Virus 35S (CaMV35S) promoter [28] when transformed into the Arabidopsis *Δppd* mutant [9]. Accession numbers of the sequences used were: *A*. *thaliana* (accession NP_567442.2), *S*. *moellendorffii* (accession XP_002964672), *A*. *trichopoda* (accession XP_006838952.1), *P*. *abies* (accession MA_99597G0010, congenie.org), *T*. *repens* (accession MZ736871) and *M*. *acuminata* (accession M0S6C8_MUSAM, Uniprotkb). Each putative PPD open reading frame (ORF) was optimised for expression in Arabidopsis [29]; this included a modified Joshi sequence [30], optimisation of codons, removal of mRNA instability sequences, removal of polyA signal sequences, removal of cryptic splice sites, addition of a *Bam*HI removable C-terminal V5 epitope and His tag tail (amino acid sequence: GGGSAKGELRGHPFEGKPIPNPLLGLDSTRTGHHHHHHGS) and addition of a double stop codon. The construct was then inserted between the CaMV35s promoter and *ocs* terminator by GATEWAY® cloning. The plant transformation vector contained a bar gene (phosphinothricin acetyl transferase) which confers resistance to the herbicide ammonium glufosinate and allowed identification of transformants under selection.

### Transformation of Arabidopsis

Gene constructs were transformed into the Arabidopsis *Δppd* mutant [9] by the floral dip infiltration method [31] and primary transformants identified following application of glufosinate-ammonium (Basta®) at 0.075 mg/ml. Independently transformed plants were confirmed by standard PCR analysis techniques using a combination of transgene-specific and T-DNA primers. Germination of hemizygous seed collected from initial transformants enabled screening for complementation of the mutant. Selected lines were analysed by Southern blot [32] and allowed to self-fertilize to create lines homozygous for the transgene.

### Immunoblot analysis

Plant tissue was disrupted using the BeadRuptor system and an extraction buffer consisting of 1:4 diluted 4× lithium dodecyl sulfate sample buffer (Life Technologies), 8 M urea, and 5% [v/v] β-mercaptoethanol. Equal quantities of protein were separated by SDS-PAGE (Mini-PROTEAN TGX Stain-Free 4–15%, Bio-Rad) and visualised for total protein with ChemiDoc™ XR S+ apparatus followed by blotting onto 0.2µm PVDF using the TransBlot® Turbo™ Transfer system (Bio-Rad). The membrane was blocked in medium containing 5% (w/v) skim milk powder in TBST (Tris-buffered saline [50 mM Tris, pH 7.4, 100 mM NaCl] + 0.1% Tween 20) for 1.5 hours at room temp (RT) or overnight at 4°C. Following 3 washes in TBST for 10min each, blots were incubated with a 1:10,000 dilution of mouse-produced anti-V5 antibody (Life Technologies) in blocking TBST as above (1 hour, RT), followed by three further washes of 10 min each in TBST. Membranes were then incubated with a 1:2,500 dilution of anti-mouse IgG horseradish peroxidase-linked whole antibody from goat (Life Technologies) in blocking TBST (1 hour, RT) before three final washes in TBST. Enzyme activity was visualized using chemiluminescence (Western Bright™ ECL-spray, Advansta) and the ChemiDoc™ XR S+ system.

### Bioinformatic analysis

The NCBI ‘nr’ protein database (GenBank Release 244.0, [https://www.ncbi.nlm.nih.gov/genbank/]) and Phytozome v13 database (https://phytozome-next.jgi.doe.gov/) were screened using the PPD domain from *Arabidopsis thaliana* PPD1 (LAKPLKLLTEEDISQLTREDCRKFLKDKGMRRPSWNKSQAIQQVLSLKALYEP) using BLASTP (version 2.10.1+, [33]) with default parameters; searches were also carried out with filtering of low complexity regions turned off. Alignments were created using the MUSCLE alignment tool [34] in the Geneious software package (Geneious Prime 2019.1.1 [https://www.geneious.com]). Synteny was calculated by pairwise genome alignments using Ensembl (http://plants.ensembl.org) [35].

## Results

### PPD is present in all vascular plants but absent from the Poaceae

**Fig 2:**
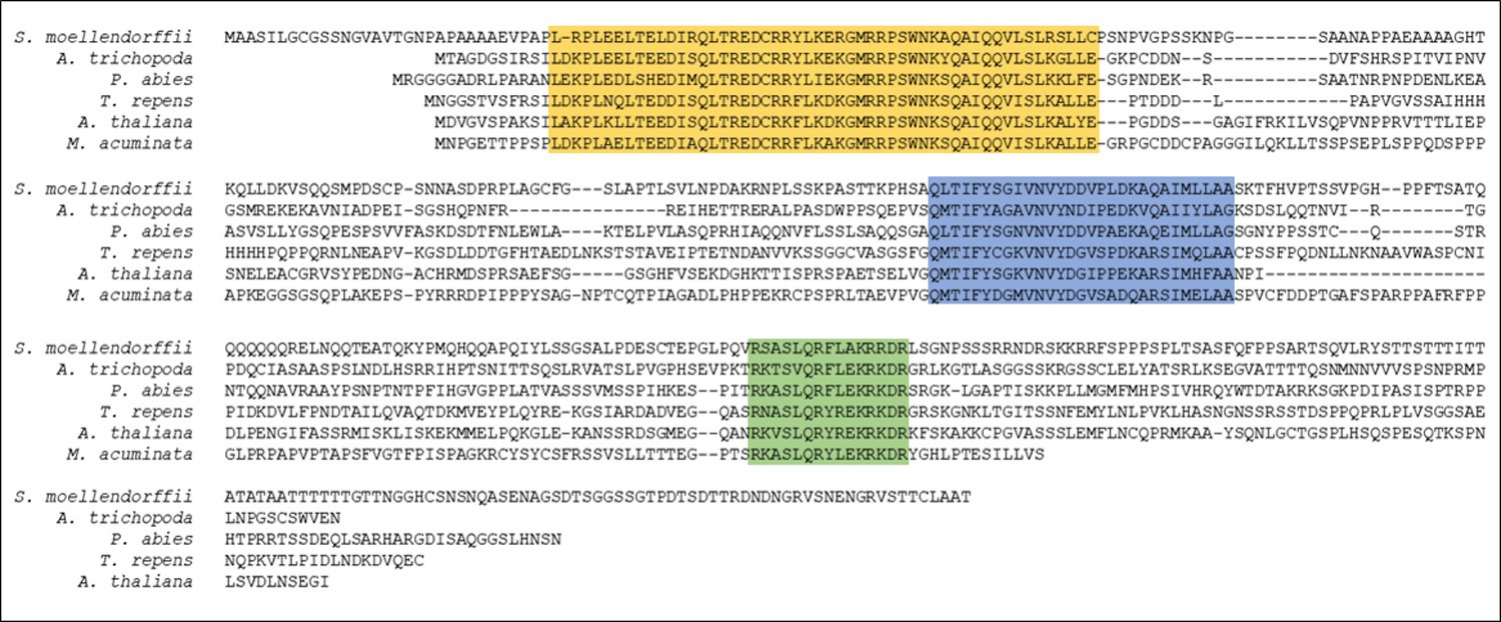
Alignment of representative PPD peptide sequences. PEAPOD proteins contain three regions that are highly conserved; these are indicated by the yellow background (PPD domain), blue background (TIFY domain) and green background (truncated Jas domain). Sequences include: *A*. *thaliana* (accession NP_567442.2), *S*. *moellendorffii* (accession XP_002964672), *A*. *trichopoda* (accession XP_006838952.1), *P*. *abies* (accession MA_99597G0010, congenie.org), *T*. *repens* (accession MZ736871) and *M*. *acuminata* (accession M0S6C8_MUSAM, Uniprotkb).

The Arabidopsis PPD domain [4] was used to search the plant genome and protein data bases; strong matches were identified in the true vascular plants including the lycophyte *S*. *moellendorffii*. In keeping with previous reports [4,9,10,12,14], there were no hits to any members of the Poaceae family. Fig 2 shows representative putative peptide sequences from *S*. *moellendorffii*, the gymnosperm *Picea abies*, basal angiosperm *A*. *trichopoda*, monocot *M*. *acuminata*, and dicot *Trifolium repens* aligned with *A*. *thaliana* PPD1. These examples all contained the three domains found in the *PPD* subfamily of *TIFY* genes as identified previously [4], including the N-terminal PPD domain (53–57 residues), an internal TIFY domain (28 residues) and a C-terminal truncated Jas domain (15 residues). The identities across the domain sequences were high, PPD (69–89%); TIFY (57–77%); and Jas (67–93%) (Table 1). While the spacing between the domains of the examples above were of similar lengths the inter-domain sequences were poorly conserved; the overall identity between the sequences in Fig 2 was 25–37%.

**Table 1:** Sequence identity across full sequences and distinct domains of PPD orthologues.

	***S*. *moellendorffii***	***A*. *trichopoda***	***P*. *abies***	***T*. *repens***	***A*. *thaliana***	***M*. *acuminata***	**Full length PPD**
***S*. *moellendorffii***		26	29	25	27	31	
***A*. *trichopoda***	26		33	30	29	32	
***P*. *abies***	29	33		27	30	33	
***T*. *repens***	25	30	27		36	37	
***A*. *thaliana***	27	29	30	36		36	
***M*. *acuminata***	31	32	33	37	36		
***S*. *moellendorffii***		78	69	73	71	70	**PPD domain**
***A*. *trichopoda***	78		75	83	81	85	
***P*. *abies***	69	75		74	75	75	
***T*. *repens***	73	83	74		87	89	
***A*. *thaliana***	71	81	75	87		83	
***M*. *acuminata***	70	85	75	89	83		
***S*. *moellendorffii***		68	86	68	64	64	**TIFY domain**
***A*. *trichopoda***	68		61	57	57	54	
***P*. *abies***	86	61		64	68	64	
***T*. *repens***	68	57	64		79	82	
***A*. *thaliana***	64	57	68	79		68	
***M*. *acuminata***	64	54	64	82	68		
***S*. *moellendorffii***		67	80	67	60	73	Jas* domain
***A*. *trichopoda***	67		87	67	73	80	
***P*. *abies***	80	87		80	80	93	
***T*. *repens***	67	67	80		87	87	
***A*. *thaliana***	60	73	80	87		87	
***M*. *acuminata***	73	80	93	87	87		

Percent identities between full length PPD sequences, PPD domains, TIFY domains and Jas* domains for *S*. *moellendorfii*, *A*. *trichopoda*, *P*. *abies*, *T*. *repens*, *A*. *thaliana* and *M*. *acuminata*.

**Fig 3:**
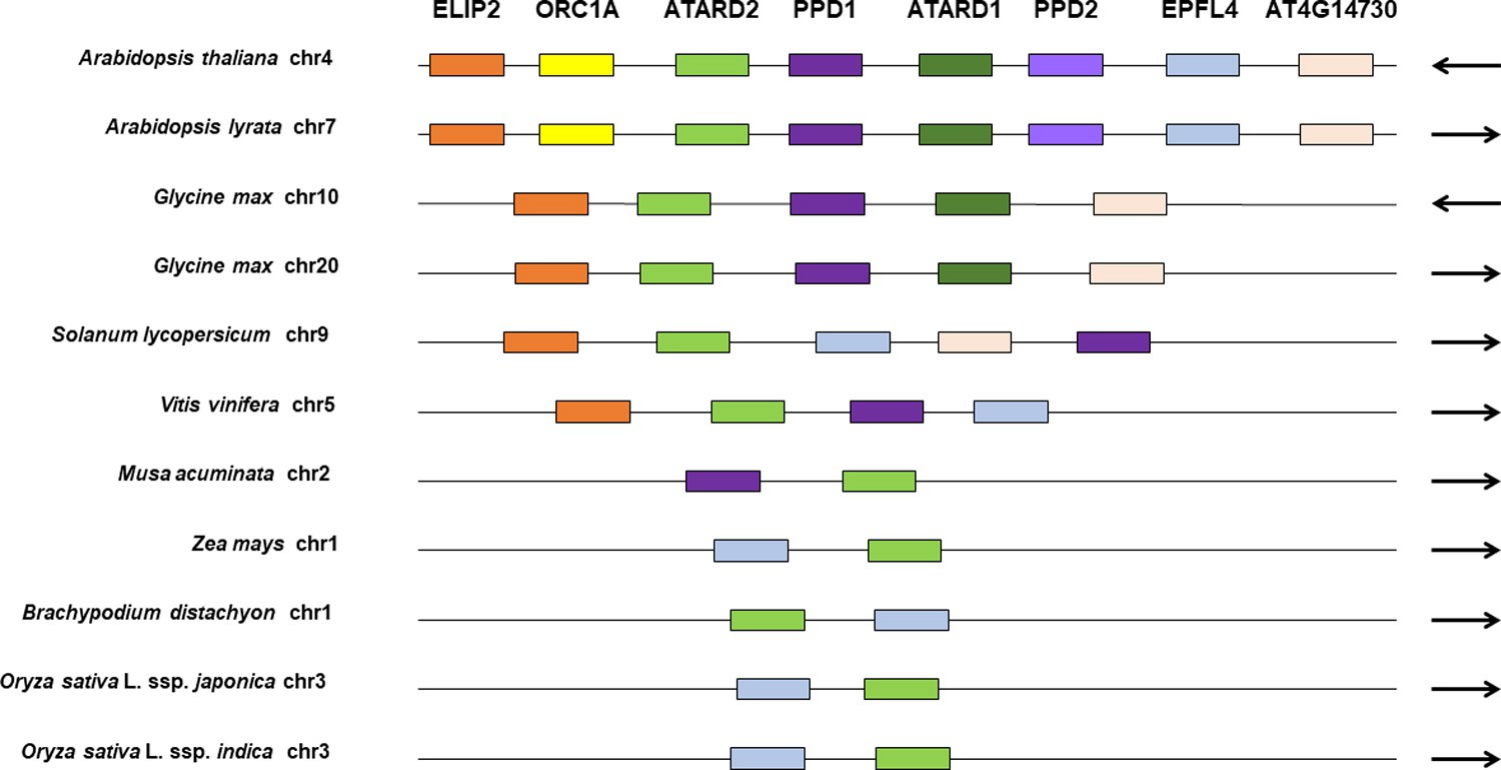
Schematic diagram illustrating syntenic comparison of PPD and flanking genes in dicotyledons and monocotyledons. Arrows indicate chromosome strand; where appropriate, the gene order has been reversed for easy graphical view. PPD1 and PPD2 are both shaded purple to signify they are very similar and in cases where only one orthologous protein was found in another organism it may represent either of the two genes. Similarly for ATARD1 and ATARD2. Only the closest syntenic clusters relative to the PPD gene are shown for *G*. *max*; *S*. *lycopersicum*, *V*. *vinifera* and *M*. *acuminata*.

To further investigate the lack of PPD in Poaceae, we performed syntenic searches using a number of flanking genes (*ELIP2*, *ORC1A*, *ATARD2*, *ATARD1*, *EPFL4*, and *AT4G14730*) in a range of genomes; including: *A*. *thaliana*, *A*. *lyrata*, *Glycine max*, *Solanum lycopersicum*, *Vitis vinifera*, *M*. *acuminata*, *Zea mays*, *Brachypodium distachyon*, *Oryza sativa* ssp. *japonica*, and *Oryza sativa* ssp. *indica*. Putative *PPD* genes and a number of the neighbouring genes were found in all the genomes except the Poaceae where only the flanking genes were found (Fig 3). Analysis of the sequence between the flanking genes in Poaceae revealed that *PPD* appears to have been replaced by a repeat rich region (S1 Fig).

### The functionality of PPD is conserved in vascular plants

**Fig 4:**
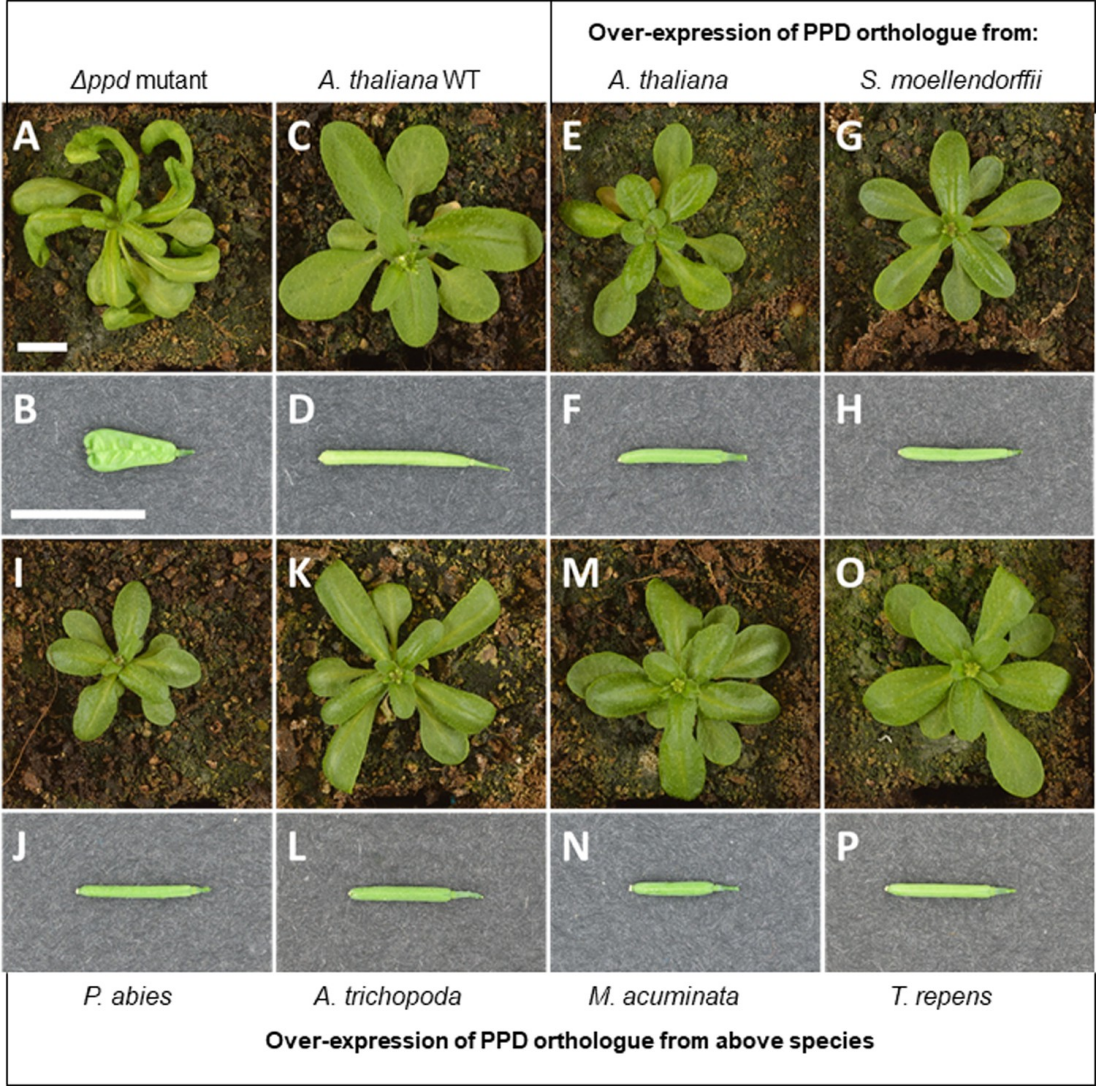
The *Arabidopsis thaliana* Δ*ppd* mutant was complemented with PPD from a broad range of multicellular land plants. In Arabidopsis the Δ*ppd* mutant has a distinct propeller shaped rosette with transversely domed leaves (A) and dimpled, short paddle shaped siliques (B); these contrast to the WT rosette of straight, transversely flat leaves (C) and smooth, elongated torpedo shaped siliques (D).Over-expression of PPD from *A*. *thaliana* (E & F), and orthologues from *S*. *moellendorffii* (G & H), *P*. *abies* (I & J), *A*. *trichopoda* (K & L), *M*. *acuminata* (M & N), and *T*. *repens* (O & P) all complemented the Δ*ppd* mutant. Plants shown are homozygous lines for each construct. Scale bars = 10mm.

**Fig 5:**
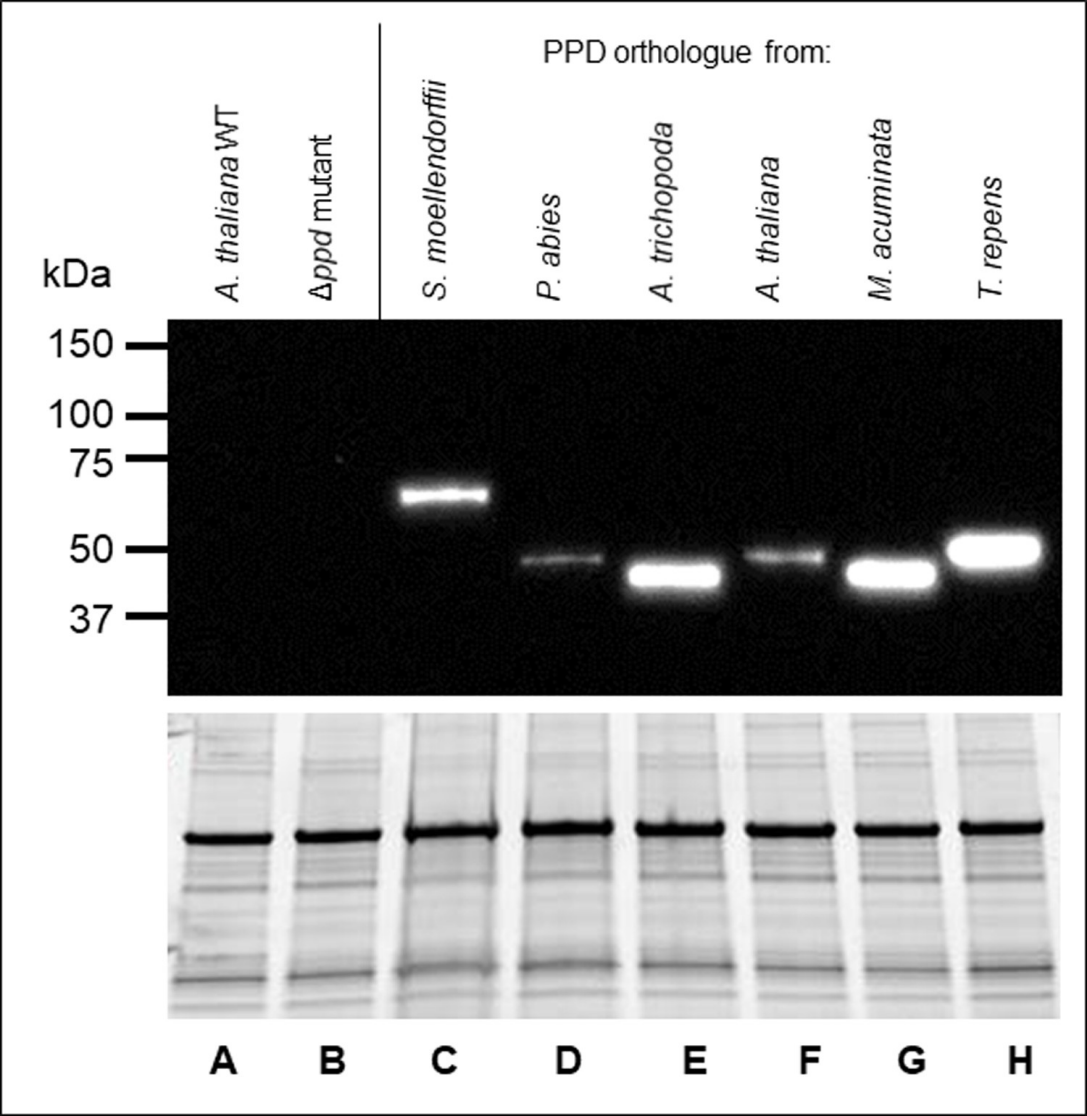
Immunoblot analysis of recombinant C-terminally V5 tagged PPDs expressed in the leaves of the *Δppd* mutant *A*. *thaliana*. Equal quantities of crude protein extract (bottom panel) from 17–19 day old rosette leaves of wild type *A*. *thaliana* (L*er*) (A); Δ*ppd* mutant (B); Δ*ppd* mutant transformed to over express the PPD orthologue from: *S*. *moellendorffii* (C); *P*. *abies* (D); *A*. *trichopoda* (E); *A*. *thaliana* (F); *M*. *acuminata* (G) and *T*. *repens* (H) were subjected to PAGE-immunoblot. The membrane was probed using the anti V5 antibody (Life Technologies). No V5 signal was detected in the WT and Δ*ppd* mutant lanes while signals of the appropriate sizes were detected in transformed Δ*ppd* mutants over-expressing a PPD orthologue (top panel).

In order to investigate the functionality of the PPD orthologues from *S*. *moellendorffii*, *P*. *abies*, *A*. *trichopoda*, *M*. *acuminata*, and *T*. *repens*, we optimised their nucleic acid ORFs and fused them to in-frame C-terminal V5-His tags. Each complete ORF was then placed under the control of a CaMV35s promoter and transformed into the *A*. *thaliana* (L*er*) *Δppd* mutant [9]. An Arabidopsis *PPD1* clone was also used to transform the mutant as a positive control and Basta®-resistant hemizygous plants were assessed visually for complementation of the mutant phenotype. The Arabidopsis Δ*ppd* mutant has transversely domed leaves in a distinct propeller-shaped rosette and short paddle-shaped siliques with raised nodes (resembling peas in a pod). In contrast, the WT has a rosette of straight, transversely flat leaves and smooth, elongated torpedo shaped siliques. Although a range of complementation levels were observed, the orthologues of *S*. *moellendorffii*, *P*. *abies*, *A*. *trichopoda*, *M*. *acuminata*, *T*. *repens* and *A*. *thaliana* were all able to complement the mutant phenotype to some extent and return *Δppd* to the rosette of straight, flat transverse profile leaves and elongated smooth siliques of the WT (Fig 4). Immunoblot analysis of crude extracts from rosette leaves of 17–19 day-old complemented Arabidopsis *Δppd* mutant lines revealed that the recombinant Arabidopsis PPD and its orthologues (from *S*. *moellendorffii*, *P*. *abies*, *A*. *trichopoda*, *M*. *acuminata*, and *T*. *repens*) were present and of the appropriate size (Fig 5). The same technique was also used to detect recombinant PPD in the flowers and siliques of complemented Δ*ppd* mutants (S2 Fig).

Complementation phenotypes ranged within each construct and between constructs. Some plants in all lines showed a partial complementation phenotype, often with the leaf exhibiting full complementation and the silique only partial complementation. The tally of fully complemented plants for each construct demonstrated the range of complementation; partial complementation was considered as not complemented (Table 2). In the lines complemented with the Arabidopsis construct, the two plants with partially complemented siliques showed only mild mutant seed pod characteristics such as slightly dimpled walls and marginally increased silique width. In constructs containing sequences from other species there was a more diverse range of silique phenotypes. For example, in *A*. *trichopoda* and *M*. *acuminata*, the least complemented siliques were very similar to the Arabidopsis *Δppd* seed pod phenotype (S3 Fig).

**Table 2:** Ratios of complemented Arabidopsis *Δppd* plants with constructs containing optimised PPD sequences from various species.

Full Complementation of:		
Species	Leaf	Silique
*A*. *thaliana*	13/13	11/13
*T*. *repens*	13/13	12/13
*P*. *abies*	5/5	3/5
*M*. *acuminata*	9/9	8/9
*A*. *trichopoda*	14/14	12/14
*S*. *moellendorffii*	19/19	14/19
*S*. *moellen* short	2/5	0/5

Ratios of complemented hemizygous Arabidopsis *Δppd* mutant plants following transformation and selection (resistance to glufosinate-ammonium) with constructs containing optimised PPD sequences from various species. Apart from the *S*. *moellendorffii* short sequence, all transformants complemented the mutant leaf phenotype and the majority of lines showed complementation of the silique phenotype of *Δppd*. Note that for silique and leaf phenotype, partial complementation was considered not complemented.

In addition to the functional PPD identified in the lycophyte *S*. *moellendorffii* (accession XP_002964672), a second potential PPD sequence was identified (accession XP_024523063) with high identity over the PPD domain and containing a TIFY and Jas* domain. This sequence was much shorter than the other *S*. *moellendorffii* sequence, with reduced spacing between the domains compared to the Arabidopsis PPD1 sequence. The sequence also contained a divergent TIFY domain compared to other species due to six extra amino acids (LFPLAY) near the C-terminus of the domain, and the sequence TMFY instead of the conserved TIFY motif (S4 Fig). The *S*. *moellendorffii* short construct did not fully complement the Arabidopsis *Δppd* mutant in any lines obtained from the screening of hemizygous plants (Table 2 and S5 Fig).

### Close relatives of the Poaceae contain orthologues of PPD

The reason for loss of PPD from the grasses has not been clarified so we set out to investigate if plants with similar growth form and stomatal arrangement to grasses contain PPD orthologues. We examined the phylogenetically closest relatives of the grasses for PPD and binding partner orthologues, to gain insight into evolution of the PPD module.

**Fig 6:**
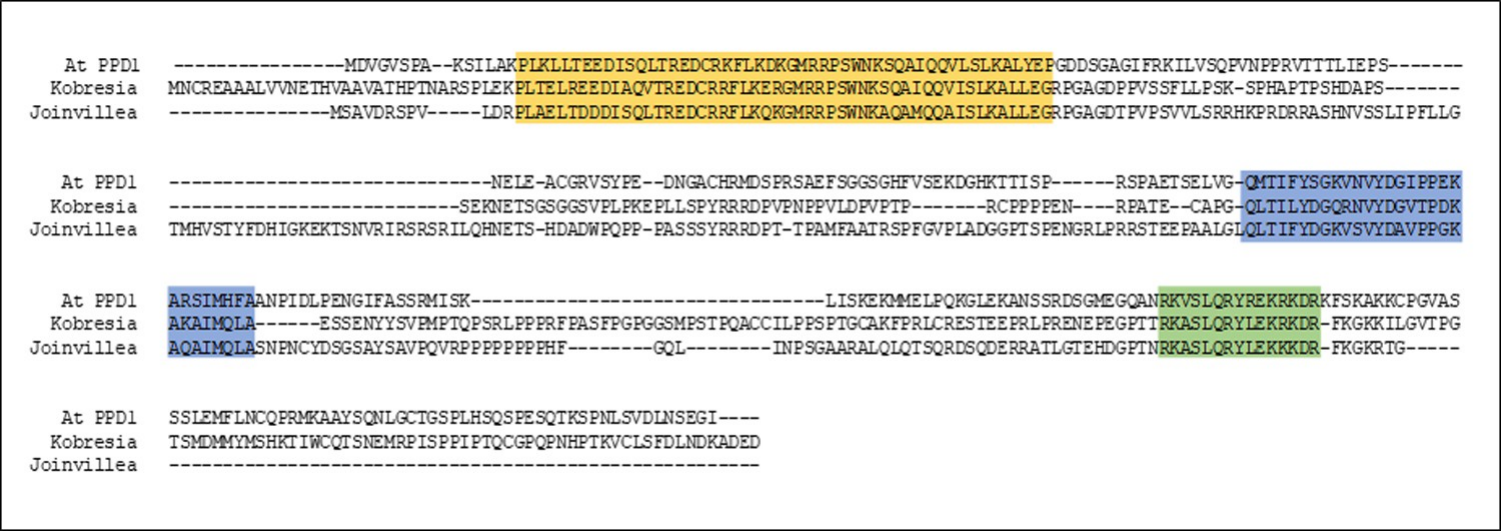
Alignment of Arabidopsis PPD1 with putative *Kobresia* and *Joinvillea* PPD proteins. The alignment shows the conserved PPD (yellow), TIFY (blue) and Jas* (green) domains.

**Fig 7:**
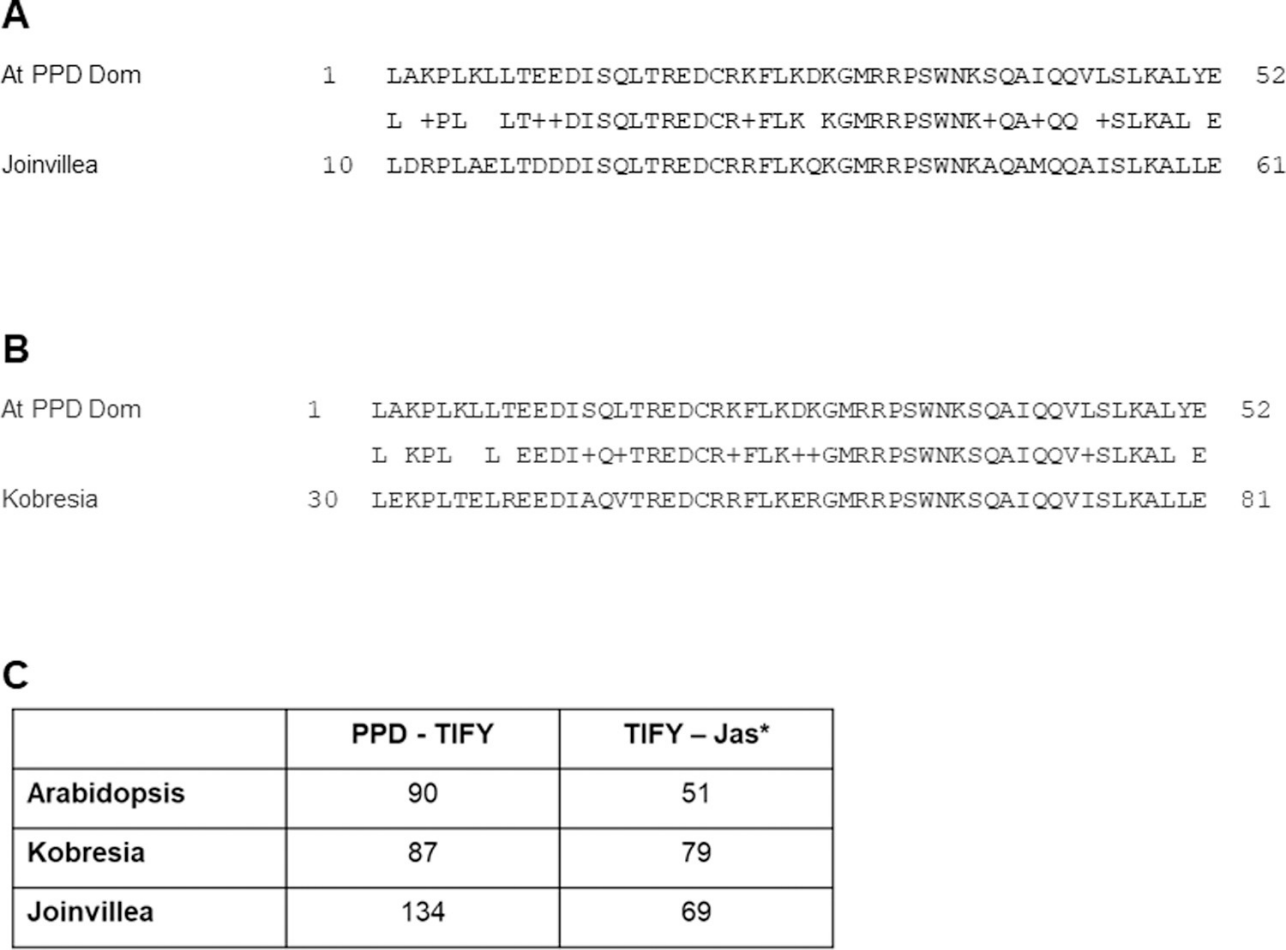
Comparison of the protein domains of putative *Joinvillea ascendens* and *Kobresia littledalei* PPDs with Arabidopsis PPD1. **A. & B.** The PPD domain from Arabidopsis aligned with putative PPD domains from *J*. *ascendens* (A) and *K*. *littledalei* (B). The *J*. *ascendens* alignment gave 75% identities and 88% positives, while *K*. *littledalei* gave 79% identities and 90% positives. **C.** The distance between domains is altered in *Kobresia* and *Joinvillea* compared to Arabidopsis, a notable feature being the greatly increased spacing between PPD and TIFY domains for the *Joinvillea* protein in contrast with the Arabidopsis sequence.

Within the order Poales, the family of Cyperaceae (sedges) includes *Kobresia littledalei*. Blast searches of this genome [36] (NCBI Genbank [37], taxid:544730) with the Arabidopsis PPD domain resulted in a potential PPD orthologue (accession KAF3322876.1) with good identity over the domain. Similarly the Joinvilleaceae (native to the Islands of Hawaii and Oceania) represent a significant evolutionary node within the Poales, as the last family to diverge before the grasses arose [38]. The reference genome of *Joinvillea ascendens* (‘Ohe’) (Phytozome, *Joinvillea ascendens* v1.1, DOE-JGI, http://phytozome-next.jgi.doe.gov/ [38]) also revealed an orthologue of PPD (accession #Joasc.01G004500.3.p) with high identity to the Arabidopsis PPD domain (Figs 6, 7A and 7B).

The *K*. *littledalei* and *J*. *ascendens* putative PPDs have increased spacing between the TIFY and Jas* domains compared to Arabidopsis but only *Joinvillea* has increased spacing between PPD and TIFY; the inter-domain spacing for these motifs is similar in *Kobresia* and Arabidopsis (Figs 6 and 7C). In the TIFY domain, the putative *Kobresia* protein contains the sequence TILY, which is divergent from the conserved TIF[F/Y]XG motif [4], however it does contain the completely conserved glycine residue and the substitute leucine is equivalent in hydrophobicity to the phenylalanine it replaces. In addition, several TIFY motif variants have been identified in Arabidopsis and *Oryza sativa* (rice) including TLF[F/Y]XG, TLL[F/Y]XG, TLS[F/Y]XG and TLV[F/Y]XG [5,39].

In Arabidopsis, PPD and KIX8/9 constitute the functional PPD module, with the regulator SAP directly interacting with PPD and KIX8/9 and targeting them for degradation by the ubiquitin ligase complex [14,15]. We identified putative proteins in the *K*. *littledalei* and *J*. *ascendens* genomes containing the KIX domain (S6A Fig). The proteins show high amino acid sequence identity at the N-terminus but this congruence is reduced towards the C-terminus. However, both putative KIX proteins contain the EAR (ETHYLENE RESPONSE FACTOR (ERF)-ASSOCIATED AMPHIPHILIC REPRESSION) motif of LXLXL in the C-terminal portion characteristic of KIX8/9 [10,13]. In Arabidopsis, this motif allows recruitment of the co-repressor TPL, thus KIX8/9 act as molecular bridges between PPD and TPL to allow negative regulation of PPD target genes [10]. A putative SAP protein, the regulator of the PPD complex, was identified in the *K*. *littledalei* genome (S6B Fig) but not in *J*. *ascendens*. The percentage identity over the entire *K*. *littledalei* SAP orthologue was not high, with 34% identities and 47% positives compared to Arabidopsis. These results indicate that the PPD module could exist in the close relatives of the Poaceae however whether the complex would carry out similar functions to those in Arabidopsis is as yet unknown.

### The PPD domain may pre-date the lycophytes

Searching current databases (Phytozome, NCBI Genbank) with the PPD domain revealed putative PPD proteins in the mosses *Sphagnum fallax* (Phytozome accessions Sphfalx04G081500.1.p; Sphfalx15G007200.1.p), *Sphagnum magellanicum* (Sphmag04G083800.4.p; Sphmag15G006400.1.p) and the liverwort *Marchantia polymorpha* (Genbank accession OAE22702.1, hypothetical protein AXG93_2675S1360). No hits were obtained for the moss *Physcomitrella patens* or the hornwort *Anthoceros angustus*. This may reflect differences in genome assembly and/or annotation, or could indicate a divergence between the bryophytes, a phylogenetic area that has yet to be fully elucidated [40].

**Fig 8:**
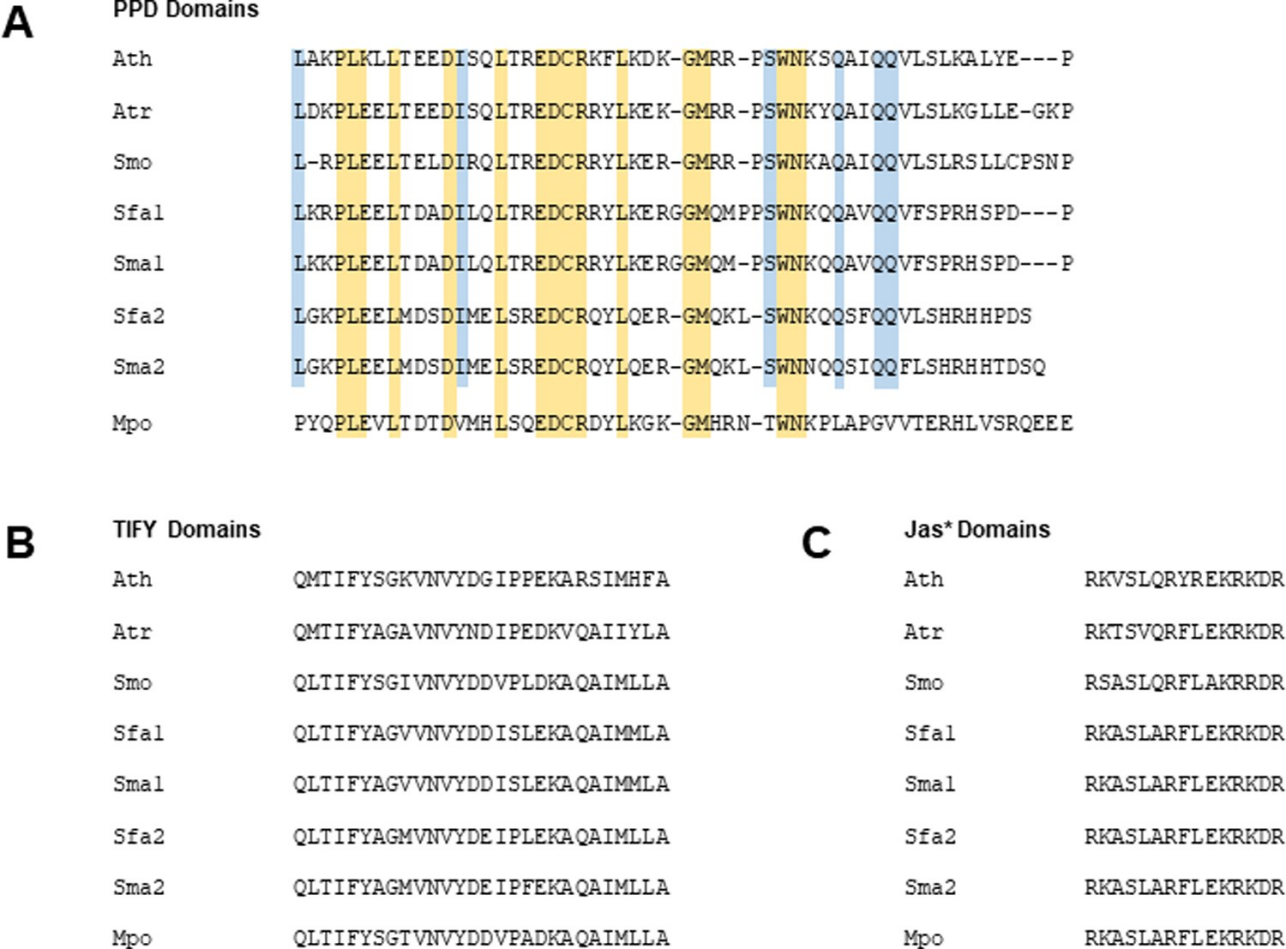
Alignment of PPD, TIFY and Jas* domains of *Arabidopsis thaliana* (Ath), *Amborella trichopoda* (Atr), *Selaginella moellendorffii* (Smo), *Sphagnum fallax* (Sfa), *Sphagnum magellanicum* (Sma) and *Marchantia polymorpha* (Mpo). **A.** Over the PPD domain, *M*. *polymorpha* is the most divergent sequence from Arabidopsis, with residues conserved in all sequences shown in yellow and residues conserved in all sequences except *M*. *polymorpha* shown in blue. **B.** The aligned TIFY domains. **C.** The aligned Jas* domains.

Across the PPD domain, the *S*. *fallax* proteins showed 55–57% identity and 75–83% positives, while the *S*. *magellanicum* proteins showed 54–59% identity and 78–80% positives (S7B Fig). The single *M*. *polymorpha* sequence had moderately low identity (38%) and positives (59%) over the PPD domain itself, although sequence similarity was higher over the other two domains (identity and positives were respectively 64% and 82% for the TIFY domain and 73% and 80% over the Jas* domain). Fig 8 shows the alignment of the PPD, TIFY and Jas* domains for *A*. *thaliana*, *A*. *trichopoda*, *S*. *moellendorffii*, *S*. *fallax*, *S*. *magellanicum* and *M*. *polymorpha*, demonstrating the level of similarity within the distinct domains.

The arrangement of the domains in the moss and liverwort putative PPDs showed substantially increased spacing compared to Arabidopsis. The moss and liverwort PPD domains are positioned approximately 100 residues upstream relative to the position of the Arabidopsis PPD domain with respect to the N-terminus and the distance between all the domains is much greater in mosses and liverworts (S7A and S8 Figs). This altered arrangement could have implications for protein function with respect to binding partner interactions.

## Discussion

The transition of life from an aquatic environment to a terrestrial setting marked an important turning point during evolution. By necessity plants preceded the transition of animals to land as a food source and following this time plants developed many additional features, such as a vascular system. Simultaneous with the move to land, plants experienced a large increase in transcription factors within each family, due to rapidly changing morphologies created by the selective pressure of new environments [3]. The PPD genes are considered part of the expansion of the TIFY gene family at this time, potentially arising with the development of true vascular tissue [4].

Grasses are thought to be the last of the flowering plants to evolve, with estimates of their divergence placed around 55–70 million years ago [41]. The loss of PPD from the Poaceae is interesting considering the widespread distribution of grasses and the importance of many species in this family as food sources for humans. Grasses appear to have deleted the PPD locus and replaced it with a repeat rich region; these genomic areas have proposed functions ranging from regulation of transcription (as the 3D structure of a genome can influence gene expression) to preserving chromosomal integrity [42].

In dicotyledonous plants deletion of *PPD* leads to enlarged leaves and wide seed pods, while over expression results in a reduction in the size of both leaves and siliques [9]. We have shown that the functionality of PPD is conserved throughout vascular plants in terms of leaf morphology and silique shape. Although the role of *PPD* in *S*. *moellendorffii*, *P*. *abies*, *A*. *trichopoda*, and *M*. *acuminata* is unknown, the ability of their respective orthologues to fully complement the Arabidopsis *Δppd* mutant indicates that they are able to bind with the same partners as the native protein in Arabidopsis. The most well-studied binding partners of PPD are the Arabidopsis proteins KIX8/9 and SAP. The absence of *PPD* and *KIX8/9* genes from the Poaceae and the similarity between the *Δppd* mutant and the *kix8 kix9* double mutant led to speculation that the PPD-KIX8/9 complex may be specific for leaf growth in the second dimension of Arabidopsis [10]. Like *KIX8/9* and *PPD*, *SAP* orthologues do not exist in the grasses; however, orthologues of other PPD binding proteins such as NINJA and LIKE HETEROCHROMATIN PROTEIN1 (LHP1) [12] do have orthologues in the Poaceae.

The closest relatives of the grasses appear to contain PPD orthologues and potentially the binding partners to make up an orthologous PPD/KIX/SAP complex. *Kobresia* and *Joinvillea* have a growth form which is very similar to grasses, with a basal meristem and vegetative tillering as a means of reproduction [43]. The stomata of these genera are also very similar to grasses as they generally have paracytic stomata with “grass-type” (dumbbell shaped) guard cells, flanked by 1 or 2 subsidiary cells with their long axis parallel to the long axis of the guard cells [43]. Despite the identification of subtle differences, such as the composition of cellulose microfibrils in sedge guard walls compared to those of grasses [44], it is likely these genera form stomata in the same way as grasses. The lack of self-renewing meristemoids in grasses has been suggested as the reason for loss of PPD in the Poaceae [10,13,45] which raises the question of the function of PPD orthologues in the close relatives of the grasses. Potentially the altered domain spacing of the *Kobresia* and *Joinvillea* putative PPDs could suggest different binding partners and therefore a dissimilar function to Arabidopsis. It is tempting to speculate that since searching the *J*. *ascendens* genome did not present a match for Arabidopsis SAP, that parts of the complex were absent from this genus before the entire complex was lost as the grasses diverged. However, the assembly and annotation of each genome, and the search parameters used, can influence the results obtained therefore more in-depth analyses would be required to unequivocally state that SAP is absent from *J*. *ascendens*.

The Poaceae have characteristic physiological traits that define them from other grass-like plants, including the structure of the inflorescence and pollen morphology [46,47]. Furthermore, a key transition at the divergence of the grasses was based around the timing of embryo development. The embryo of grasses is more advanced in its development before seed release than other monocots. Precursors of vascular structures, cotyledon, leaves and root meristem are created before seed shed in grasses, while non-grass monocots don’t begin to develop these structures until after seed shed [41]. Another important difference between grasses and their next of kin is the fruit of grass plants, a unique structure amongst angiosperms called the caryopsis (grain). The relatives of grasses form ovaries made of three fused carpels, each containing a locule with an ovule, of which only one develops and the other two terminate. In contrast, the grasses only ever develop one ovule within one locule [41]. These unique features of grasses have the potential to provide clues to the loss of PPD from the Poaceae, considering the role of PPD genes in seed and fruit development [18]. Alternatively, the explanation may be due to functions that have yet to be elucidated for this complex.

The PPD domain is proposed to have formed alongside the vascular system of land plants before the divergence of lycophytes (e.g. *S*. *moellendorffii*) [4]. The continuing publication of new genome sequences has allowed us to identify putative PPD orthologues in selected bryophytes, thus hinting at more ancient origins for this gene. Bryophytes lack true vascular structures; liverworts and mosses have water conducting cells devoid of lignin while in hornworts there is an absence of any type of water conveying cells [48]. These features suggest PPD orthologues in these species have roles that differ from those in other land plants.

The hypothetical *Marchantia* PPD has less identity over the PPD domain than the two *Sphagnum* species and the possibility remains that this protein sequence encodes a non-PPD TIFY protein. Alternatively, the sequence dissimilarity could reflect evolutionary divergence, with the *Marchantia* sequence encoding a less evolved version of PPD. While the phylogeny of the bryophytes is an area of ongoing debate [40,49], one perspective recognises liverworts as the earliest diverging land plant according to morphology [50]. In plant evolution, stomata are considered one of the primary advances [51] and appeared in land plants before vasculature [40]. The sporophyte (diploid phase of the life cycle) of mosses and hornworts have stomata [51], while the gametophyte (haploid phase) of Marchantia has air pores which are thought to carry out similar functions to stomata, although do not have the ability to change the aperture size in the same way as stomata [50]. Identification of several genes controlling air pore development in Marchantia were not orthologous with any stomatal development genes, suggesting these structures are not derived from a common ancestor [52]. Our database searches were unable to identify PPD orthologues in the hornwort *Anthoceros angustus* or the moss *Physcomitrella patens*. The significance of identifying putative PPD orthologues in some bryophyte species and the absence in others is difficult to evaluate and requires further investigation. All the orthologous proteins discussed are the result of conceptual translation and variation in transcription start site prediction between sequencing projects may explain some of the inconsistency. Transformation of the Δ*ppd* mutant with the bryophyte orthologues could provide interesting information on functionality, in addition to creating mutants of putative *PPD* genes in *Marchantia* and *Sphagnum*. As the range of sequenced bryophyte genomes increases and annotation of genomic data is enhanced, new information will be available to elucidate the evolution of PPD.

## Conclusion

Despite the highly conserved domain sequence and function of PPD throughout rosid and asterid plants, until now it was not known if the PPD orthologues in other clades encoded functional proteins. The constitutive expression of these orthologues in the Arabidopsis Δ*ppd* mutant described here demonstrates the conserved functionality of PPD throughout vascular plants. Whether this functionality extends to non-vascular plants is unknown however the orthologues identified in nominated bryophytes suggest that additional research will demonstrate new roles for PPD. Identification of PPD orthologues in the close relatives of the grasses has shed light on mechanisms which may differ between the Poaceae and physiologically similar plants, although further investigation is required to determine the reason for the intriguing absence of the complex from the grasses.

## Supporting information

S1 Fig *PPD* appears to have been replaced by a repeat rich region in the Poaceae. Syntenic comparisons of the Poaceae genomes analysed (*Brachypodium distachyon*, *Oryza sativa* and *Zea mays*) demonstrated that the region expected to contain the PPD genes had been disrupted and contained numerous repeats. Shown above is the presence of abundant repeat elements in the *Oryza sativa* chromosome where synteny predicts PPD ought to be.
(TIF)

S2 Fig Immunoblot analysis of recombinant C-terminally V5 tagged PPDs expressed in the flowers and siliques of the *Δppd* mutant *A*. *thaliana*. Equal quantities of crude protein extract from developing flowers (petals not yet visible) and the 10 most apical green siliques on the primary stalk of wild type *A*. *thaliana* (L*er*); Δ*ppd* mutant; Δ*ppd* mutant lines transformed to over express the PPD orthologues from *A*. *trichopoda*; *A*. *thaliana*; and *M*. *acuminata* were subjected to PAGE-immunoblot. The membranes were probed using the anti V5 antibody (Life Technologies). No V5 signal was detected in the WT and Δ*ppd* mutant lanes while signals of the appropriate sizes were detected in both flowers and siliques of most Δ*ppd* mutant lines transformed to over express a PPD orthologue.
(TIF)

S3 Fig Complementation of Arabidopsis *Δppd* mutant with optimised constructs from *A*. *thaliana* (C), *P*. *abies* (D), *A*. *trichopoda* (E), *T*. *repens* (F) and *M*. *acuminata* (G). WT Landsberg *erecta* and *Δppd* mutant are shown in A and B, respectively. The range of complementation of silique phenotype can be seen for each construct, varying from full correction of the mutant to WT phenotype, to siliques that appear mutant in phenotype.
(TIF)

S4 Fig Comparison of the two *S*. *moellendorffii* sequences transformed into *A*. *thaliana* Δ*ppd*. **A.** Alignment of PPD1 from Arabidopsis with putative PPDs from *S*. *moellendorffii*. PPD domain is highlighted yellow, TIFY domain blue and Jas* domain green. The TIFY domain of *S*. *moellendorffii* short (*S*. *moellen* short) contains six additional amino acids compared to the sequences of other species. **B.** Both *S*. *moellendorffii* proteins show high identity over the PPD domain with 67% identities and 88% positives for *S*. *moellendorffii* and 69% identities and 86% positives for *S*. *moellen* short. **C.** Spacing between the PPD and TIFY domains is greatly reduced for *S*. *moellen* short compared to Arabidopsis and *S*. *moellendorffii*. Both *S*. *moellendorffii* proteins show reduced spacing between TIFY-Jas* domains compared to Arabidopsis.
(TIF)

S5 Fig Arabidopsis *Δppd* mutant transformed with *S*. *moellendorffii* constructs. **A. and B**. Arabidopsis *Δppd* mutant and WT, respectively. **C.** The *S*. *moellendorffii* short sequence (SmoS) did not complement the mutant in 3/5 lines for leaf and 5/5 for silique. **D.** Examples of complementation lines containing the *S*. *moellendorffii* sequence (Smo); this construct was able to complement the leaf phenotype in 19/19 lines and the silique phenotype in 14/19 lines.
(TIF)

S6 Fig Alignment of Arabidopsis KIX8/9 and SAP proteins with putative orthologues from *K*. *littledalei* and *J*. *ascendens*. **A**. Alignment of KIX8 and KIX9 from Arabidopsis (NP_001327395 and NP_680756, respectively) with putative KIX proteins (KAF3332083.1 [NCBI] and Joasc.12G122500.1.p [JGI—Phytozome) from *K*. *littledalei* (Kl) and *J*. *ascendens* (Ja), respectively. There is high sequence identity over the N-terminal area and the conserved EAR motif in the C-terminal portion (purple box). **B.** Alignment of putative SAP (KAF3333973.1) from *K*. *littledalei* with SAP protein (NP_198426) from Arabidopsis.
(TIF)

S7 Fig Analysis of bryophyte putative PPD orthologues. **A.** Distances between domains for Arabidopsis PPD1 compared to the putative PPD proteins found in selected bryophytes. The distance between the PPD and TIFY domains is approximately double in the bryophyte species compared to Arabidopsis while the distance between TIFY and Jas* domains in Arabidopsis is nearly two thirds that of the bryophytes. **B.** Alignments of PPD domains for *S*. *fallax*, *S*. *magellanicum* and *M*. *polymorpha* putative PPD proteins with the PPD domain of Arabidopsis PPD1, showing identical and positive residues.
(TIF)

S8 Fig Alignment of putative PPD protein sequences from *Marchantia polymorpha*, *Sphagnum fallax* and *Sphagnum magellanicum* with Arabidopsis PPD1 (Accession # NP_567442.2). Conserved domains are shown as PPD domain (yellow), TIFY domain (blue) and Jas* domain (green).
(TIF)

S1 Raw images (PDF)
