# Supplementary figures and images for: Evolution and conserved functionality of organ size and shape regulator PEAPOD
Source: PLoS One. 2022 Feb 11;17(2):e0263928. doi: 10.1371/journal.pone.0263928 (PMC8836299; doi:10.1371/journal.pone.0263928)

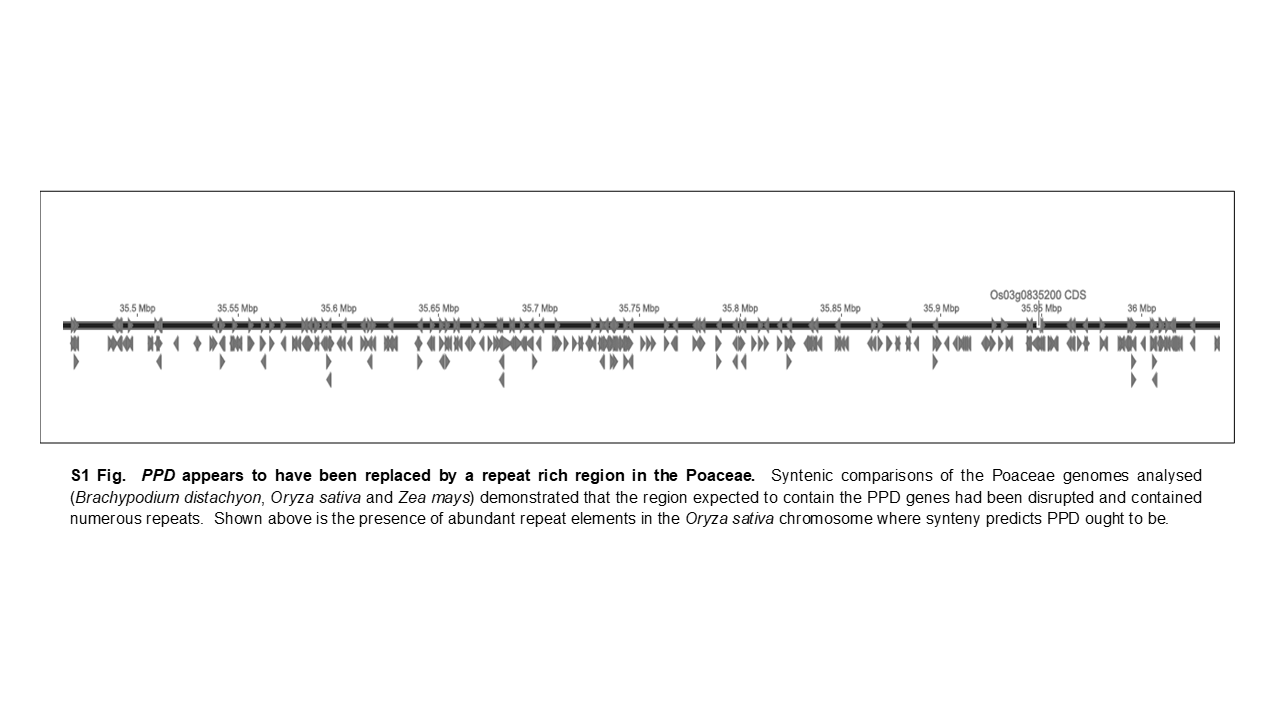

Supplement: S1 Fig — Syntenic comparisons of the Poaceae genomes analysed (Brachypodium distachyon, Oryza sativa and Zea mays) demonstrated that the region expected to contain the PPD genes had been disrupted and contained numerous repeats. Shown above is the presence of abundant repeat elements in the Oryza sativa chromosome where synteny predicts PPD ought to be. (TIF) [file pone.0263928.s001.tif]

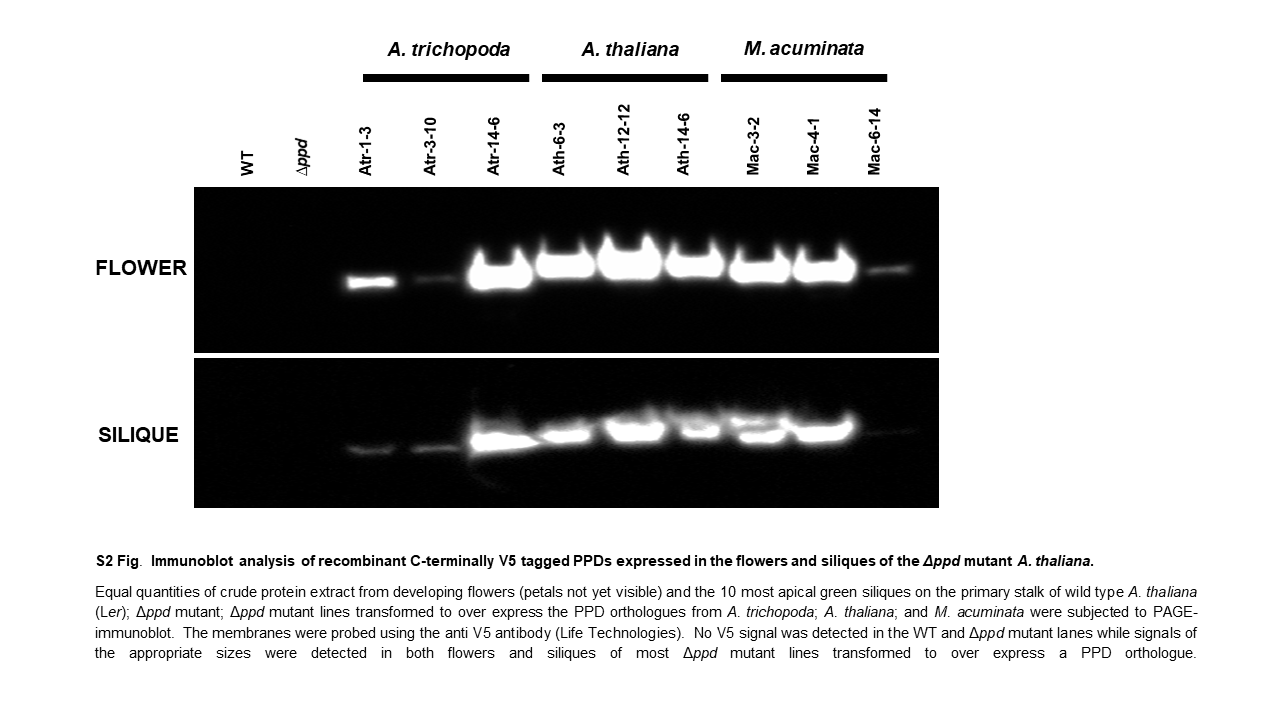

Supplement: S2 Fig — Equal quantities of crude protein extract from developing flowers (petals not yet visible) and the 10 most apical green siliques on the primary stalk of wild type A. thaliana (Ler); Δppd mutant; Δppd mutant lines transformed to over express the PPD orthologues from A. trichopoda; A. thaliana; and M. acuminata were subjected to PAGE-immunoblot. The membranes were probed using the anti V5 antibody (Life Technologies). No V5 signal was detected in the WT and Δppd mutant lanes while signals of the appropriate sizes were detected in both flowers and siliques of most Δppd mutant lines transformed to over express a PPD orthologue. (TIF) [file pone.0263928.s002.tif]

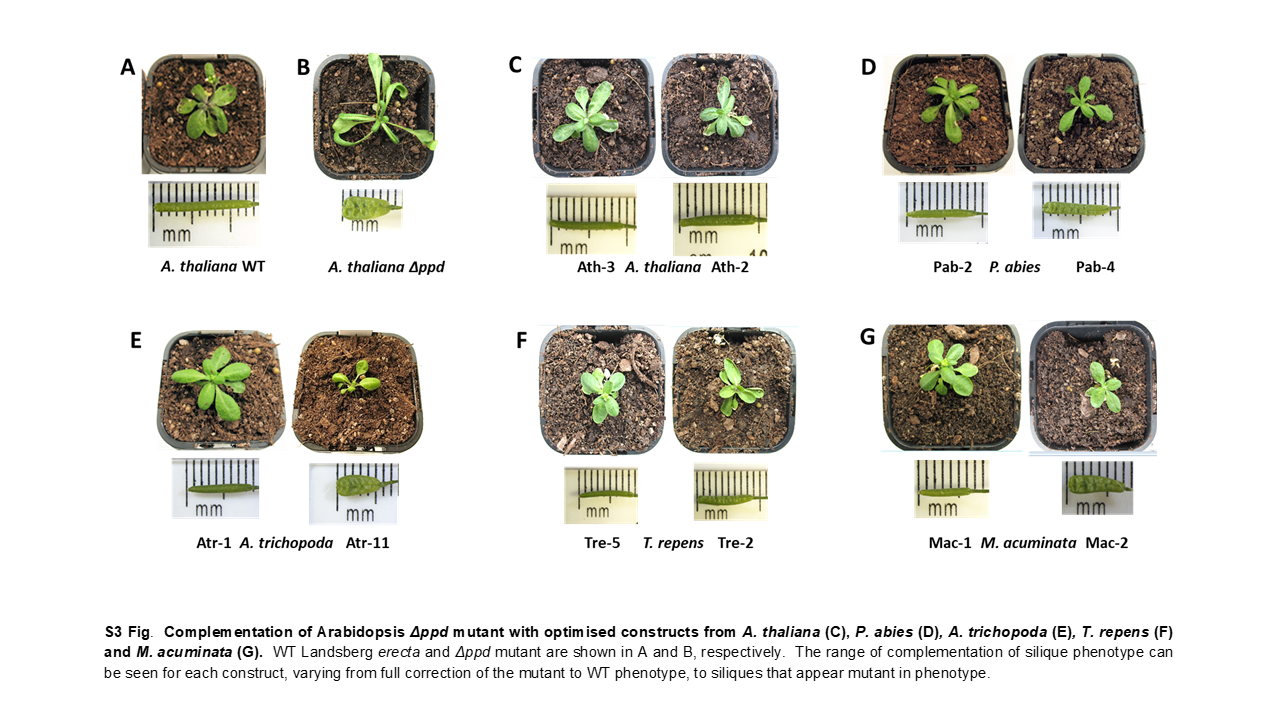

Supplement: S3 Fig — Complementation of Arabidopsis Δppd mutant with optimised constructs from A. thaliana (C), P. abies (D), A. trichopoda (E), T. repens (F) and M. acuminata (G). WT Landsberg erecta and Δppd mutant are shown in A and B, respectively. The range of complementation of silique phenotype can be seen for each construct, varying from full correction of the mutant to WT phenotype, to siliques that appear mutant in phenotype. (TIF) [file pone.0263928.s003.tif]

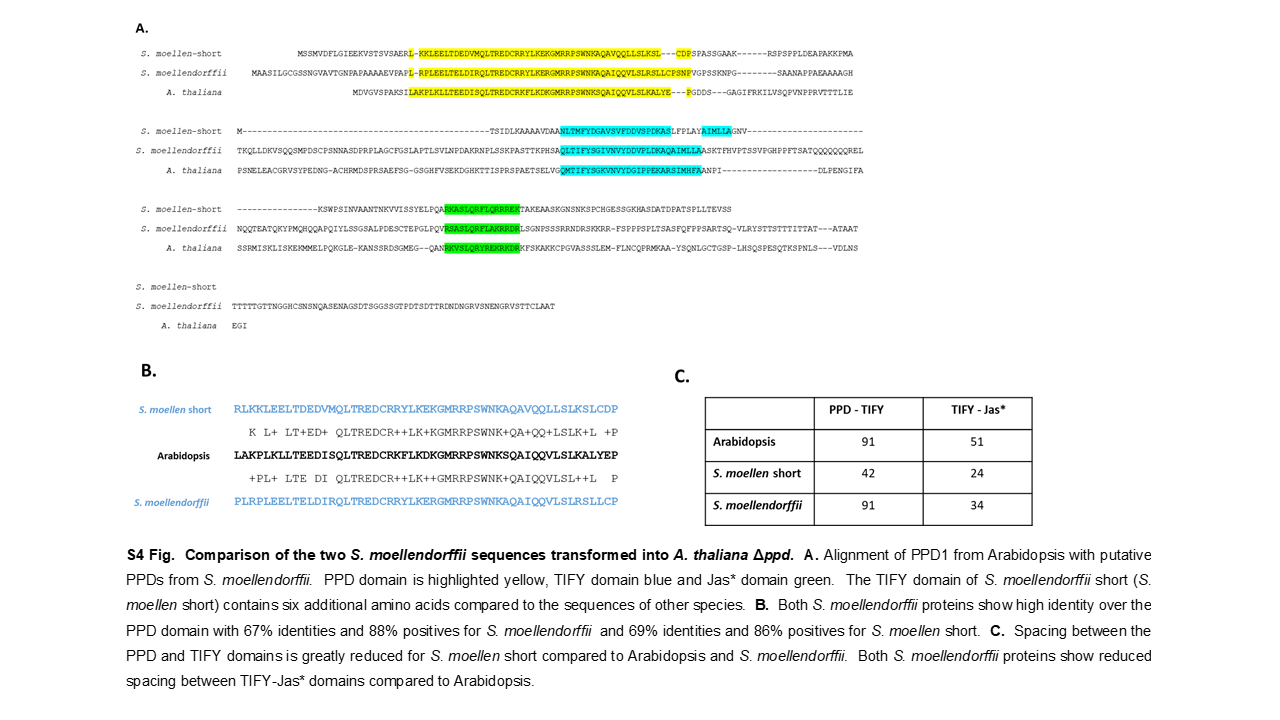

Supplement: S4 Fig — A. Alignment of PPD1 from Arabidopsis with putative PPDs from S. moellendorffii. PPD domain is highlighted yellow, TIFY domain blue and Jas* domain green. The TIFY domain of S. moellendorffii short (S. moellen short) contains six additional amino acids compared to the sequences of other species. B. Both S. moellendorffii proteins show high identity over the PPD domain with 67% identities and 88% positives for S. moellendorffii and 69% identities and 86% positives for S. moellen short. C. Spacing between the PPD and TIFY domains is greatly reduced for S. moellen short compared to Arabidopsis and S. moellendorffii. Both S. moellendorffii proteins show reduced spacing between TIFY-Jas* domains compared to Arabidopsis. (TIF) [file pone.0263928.s004.tif]

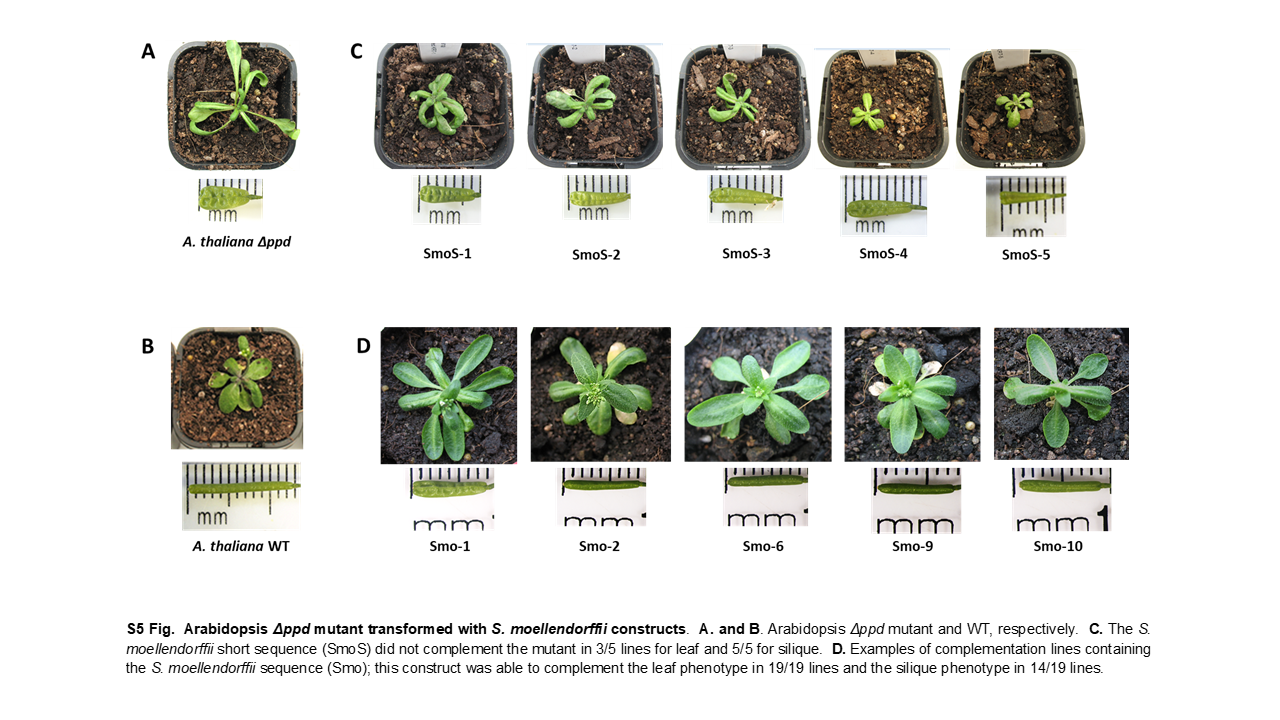

Supplement: S5 Fig — A. and B. Arabidopsis Δppd mutant and WT, respectively. C. The S. moellendorffii short sequence (SmoS) did not complement the mutant in 3/5 lines for leaf and 5/5 for silique. D. Examples of complementation lines containing the S. moellendorffii sequence (Smo); this construct was able to complement the leaf phenotype in 19/19 lines and the silique phenotype in 14/19 lines. (TIF) [file pone.0263928.s005.tif]

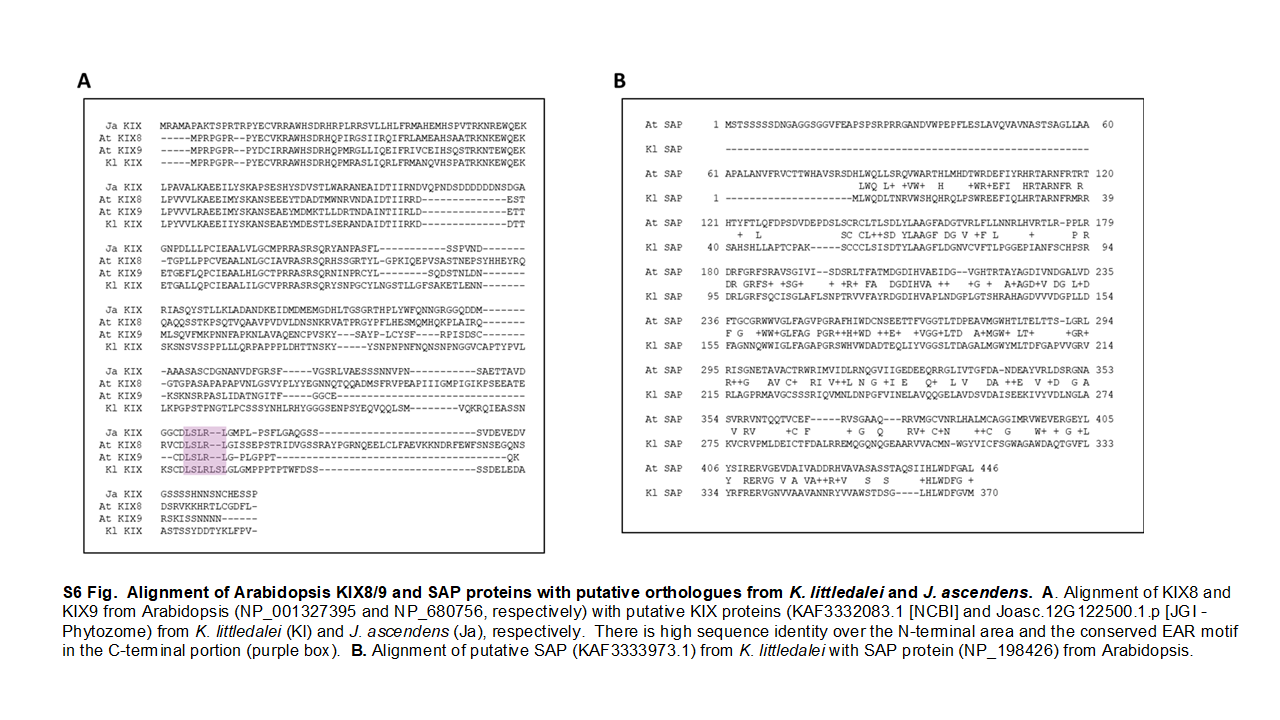

Supplement: S6 Fig — A. Alignment of KIX8 and KIX9 from Arabidopsis (NP_001327395 and NP_680756, respectively) with putative KIX proteins (KAF3332083.1 [NCBI] and Joasc.12G122500.1.p [JGI—Phytozome) from K. littledalei (Kl) and J. ascendens (Ja), respectively. There is high sequence identity over the N-terminal area and the conserved EAR motif in the C-terminal portion (purple box). B. Alignment of putative SAP (KAF3333973.1) from K. littledalei with SAP protein (NP_198426) from Arabidopsis. (TIF) [file pone.0263928.s006.tif]

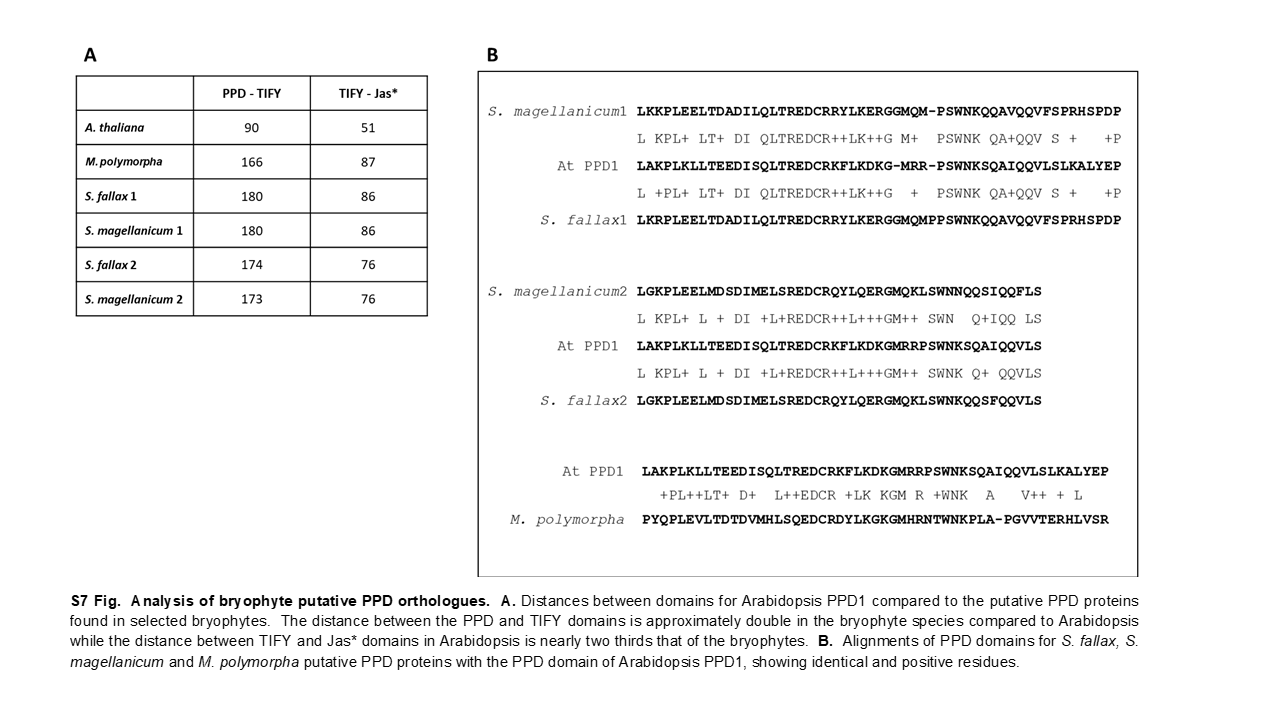

Supplement: S7 Fig — A. Distances between domains for Arabidopsis PPD1 compared to the putative PPD proteins found in selected bryophytes. The distance between the PPD and TIFY domains is approximately double in the bryophyte species compared to Arabidopsis while the distance between TIFY and Jas* domains in Arabidopsis is nearly two thirds that of the bryophytes. B. Alignments of PPD domains for S. fallax, S. magellanicum and M. polymorpha putative PPD proteins with the PPD domain of Arabidopsis PPD1, showing identical and positive residues. (TIF) [file pone.0263928.s007.tif]

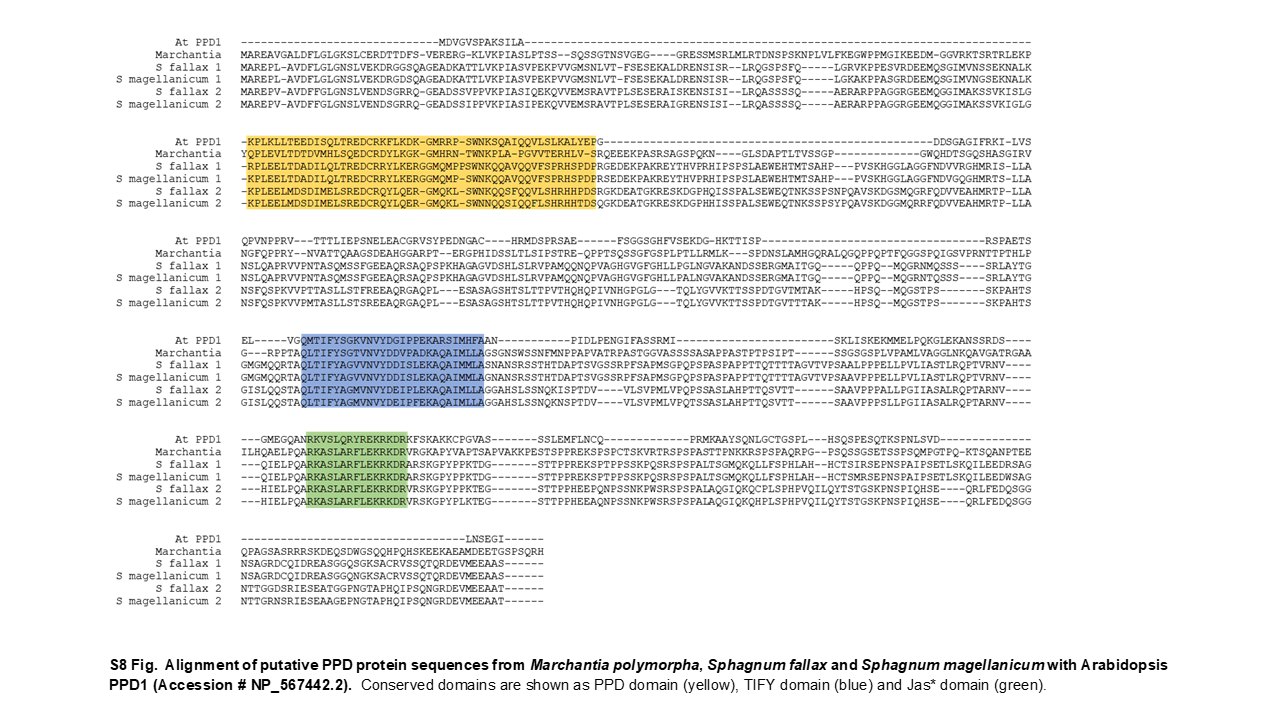

Supplement: S8 Fig — Conserved domains are shown as PPD domain (yellow), TIFY domain (blue) and Jas* domain (green). (TIF) [file pone.0263928.s008.tif]
