# Supplementary material for: Evolution and conserved functionality of organ size and shape regulator PEAPOD
Source: PLoS One. 2022 Feb 11;17(2):e0263928. doi: 10.1371/journal.pone.0263928 (PMC8836299; doi:10.1371/journal.pone.0263928)
Supplement: S1 Raw images — (PDF) [file pone.0263928.s009.pdf]

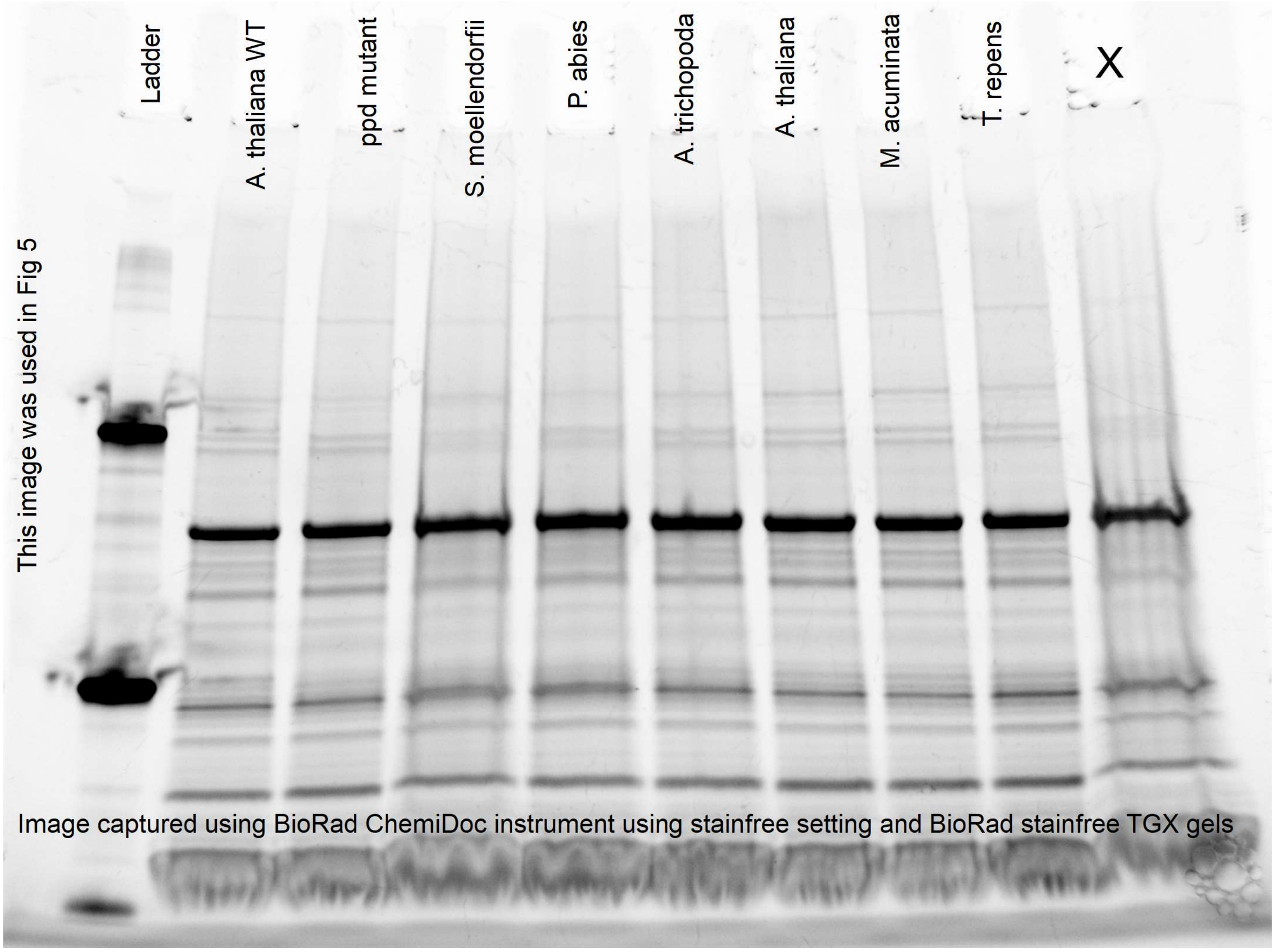

This image was used in Fig 5
Ladder
A. thaliana WT
ppd mutant
S. moellendorfii
P. abies
A. trichopoda
A. thaliana
M. acuminata
T. repens
X
Image captured using BioRad ChemiDoc instrument using stainfree setting and BioRad stainfree TGX gels

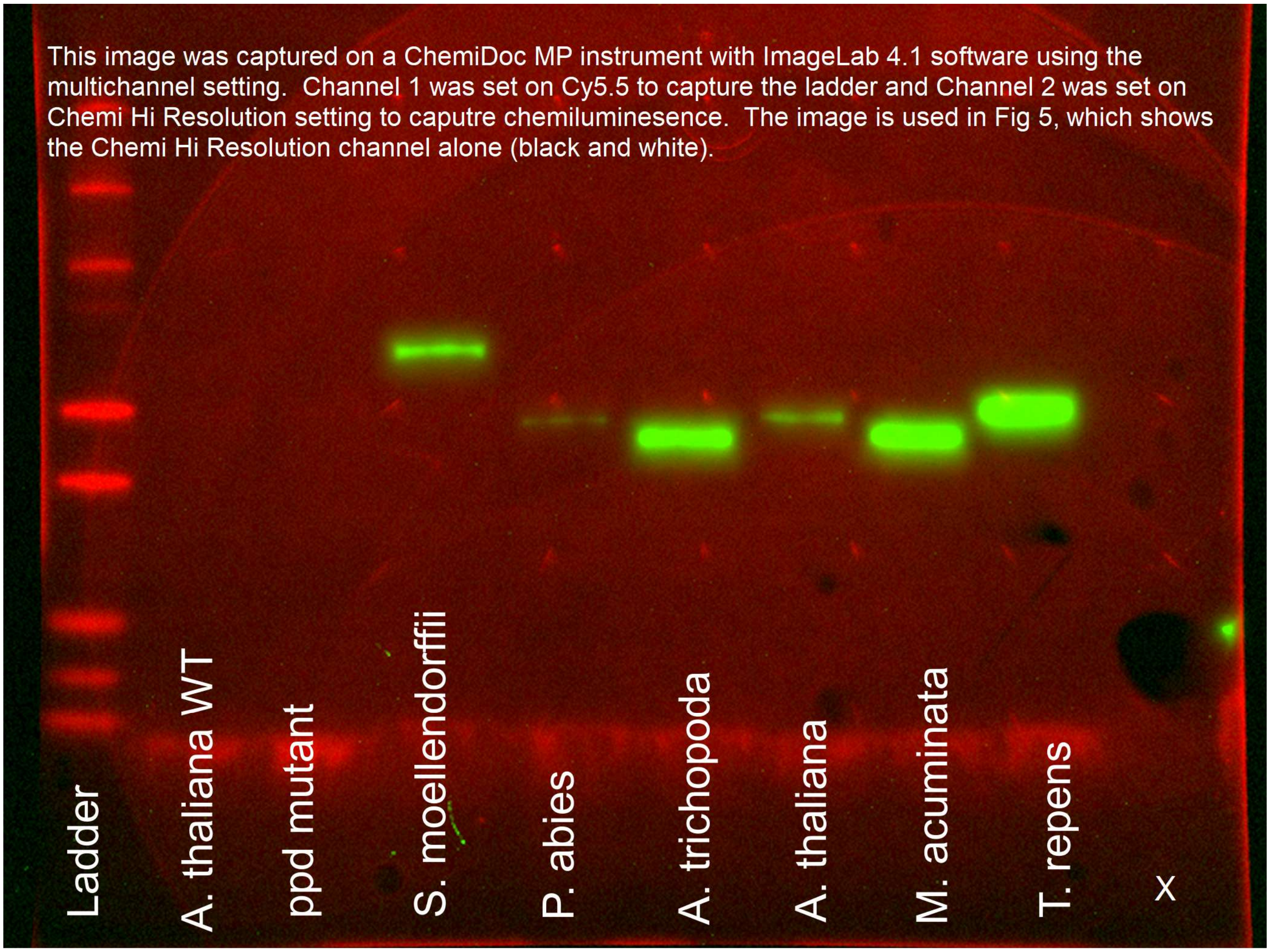
This image was captured on a ChemiDoc MP instrument with ImageLab 4.1 software using the multichannel setting. Channel 1 was set on Cy5.5 to capture the ladder and Channel 2 was set on Chemi Hi Resolution setting to caputre chemiluminesence. The image is used in Fig 5, which shows the Chemi Hi Resolution channel alone (black and white).
Ladder
A. thaliana WT
ppd mutant
S. moellendorffii
P. abies
A. trichopoda
A. thaliana
M. acuminata
T. repens
X

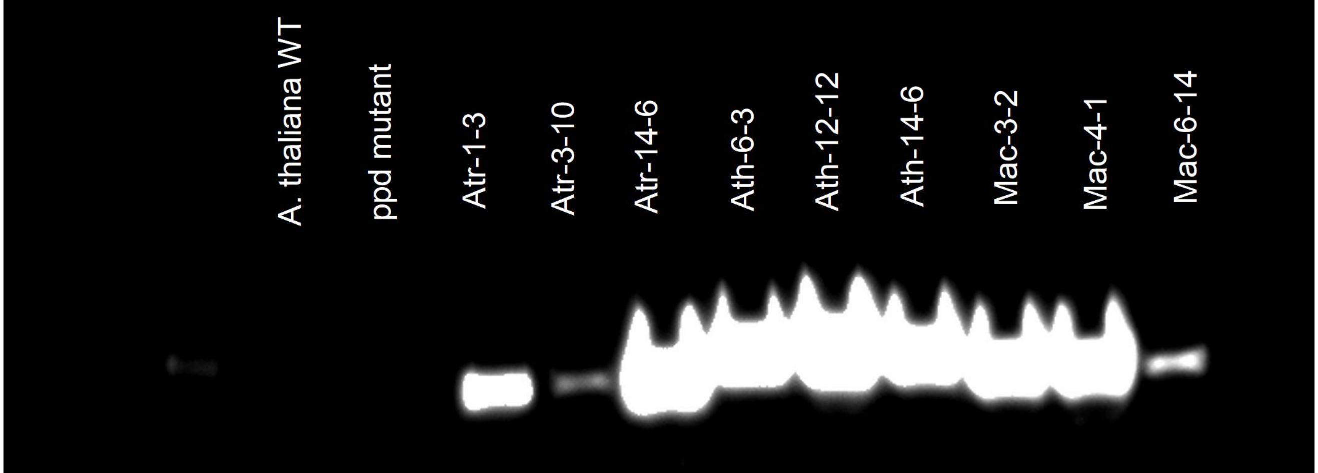

The image was captured using a BioRad ChemiDoc MP intsrument with ImageLab 4.1 software. This image was used in Supplementary Figure 2 and shows the Chemi Hi Resolution channel which captures chemiluminesence.

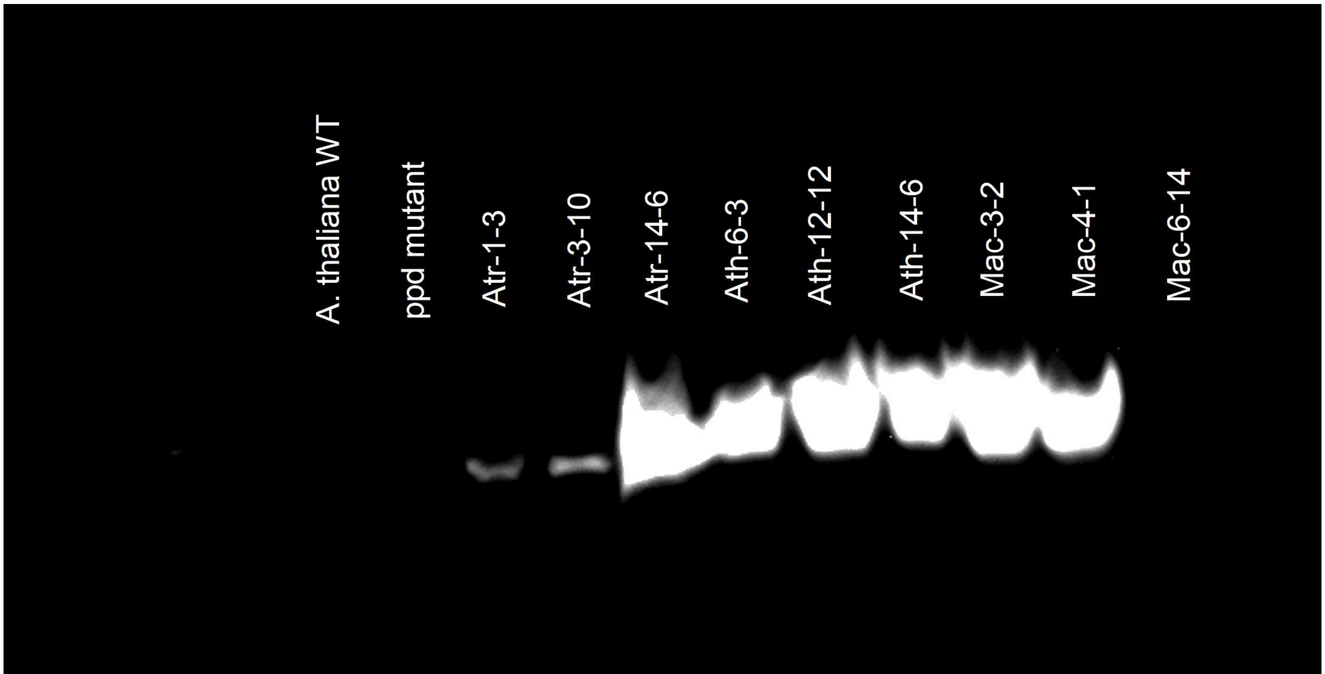

This image was captured using a BioRad ChemiDoc MP and ImageLab 4.1 software. The image was used in Supplementary Figure 2 and shows the Chemi Hi Resolution channel which captures chemiluminesence.
